# Oxidative stress induces mitochondrial iron overload and ferroptotic cell death

**DOI:** 10.1038/s41598-023-42760-4

**Published:** 2023-09-19

**Authors:** Yi Chen, Xiaoyun Guo, Yachang Zeng, Xiaoliang Mo, Siqi Hong, Hui He, Jing Li, Sulail Fatima, Qinghang Liu

**Affiliations:** Department of Physiology and Biophysics, School of Medicine, University of Washington, 1705 NE Pacific Street, G424, Box 357290, Seattle, WA 98195-7290 USA

**Keywords:** Cell death, Cell signalling

## Abstract

Oxidative stress has been shown to induce cell death in a wide range of human diseases including cardiac ischemia/reperfusion injury, drug induced cardiotoxicity, and heart failure. However, the mechanism of cell death induced by oxidative stress remains incompletely understood. Here we provide new evidence that oxidative stress primarily induces ferroptosis, but not apoptosis, necroptosis, or mitochondria-mediated necrosis, in cardiomyocytes. Intriguingly, oxidative stress induced by organic oxidants such as tert-butyl hydroperoxide (tBHP) and cumene hydroperoxide (CHP), but not hydrogen peroxide (H_2_O_2_), promoted glutathione depletion and glutathione peroxidase 4 (GPX4) degradation in cardiomyocytes, leading to increased lipid peroxidation. Moreover, elevated oxidative stress is also linked to labile iron overload through downregulation of the transcription suppressor BTB and CNC homology 1 (Bach1), upregulation of heme oxygenase 1 (HO-1) expression, and enhanced iron release via heme degradation. Strikingly, oxidative stress also promoted HO-1 translocation to mitochondria, leading to mitochondrial iron overload and lipid reactive oxygen species (ROS) accumulation. Targeted inhibition of mitochondrial iron overload or ROS accumulation, by overexpressing mitochondrial ferritin (FTMT) or mitochondrial catalase (mCAT), respectively, markedly inhibited oxidative stress-induced ferroptosis. The levels of mitochondrial iron and lipid peroxides were also markedly increased in cardiomyocytes subjected to simulated ischemia and reperfusion (sI/R) or the chemotherapeutic agent doxorubicin (DOX). Overexpressing FTMT or mCAT effectively prevented cardiomyocyte death induced by sI/R or DOX. Taken together, oxidative stress induced by organic oxidants but not H_2_O_2_ primarily triggers ferroptotic cell death in cardiomyocyte through GPX4 and Bach1/HO-1 dependent mechanisms. Our results also reveal mitochondrial iron overload via HO-1 mitochondrial translocation as a key mechanism as well as a potential molecular target for oxidative stress-induced ferroptosis in cardiomyocytes.

## Introduction

Loss of cardiomyocytes by apoptotic and/or necrotic cell death contributes to the pathogenesis of multiple forms of heart disease such as ischemia/reperfusion injury (I/R), myocarditis, cardiomyopathy, drug induced cardiotoxicity, and heart failure of diverse etiologies^1, 2^. Apoptosis has been well established as a form of regulated cell death, which is tightly regulated by death receptor- or mitochondria-mediated signaling pathways^1^. In contrast, necrosis had long been regarded as an unregulated and passive process, characterized by cellular swelling, plasma membrane rupture, and cell lyses^3^. However, recent studies have overturned this notion and revealed that necrosis can also occur in a highly regulated and genetically controlled manner, termed “regulated necrosis”^1^. Indeed, several regulated necrosis pathways have recently been identified, including necroptosis, mitochondria-mediated necrosis, ferroptosis, pyroptosis, and other regulated necrotic processes^1^.

Necroptosis is a form of regulated necrosis mediated by death receptors and executed through the induction of receptor-interacting protein kinase 1 and 3 (RIPK1-RIPK3) necrosome, phosphorylation and oligomerization of mixed lineage kinase domain-like protein (MLKL), and plasma membrane disruption^4–6^. In contrast to necroptosis, the defining event in the mitochondria-mediated necrosis is the opening of the mitochondrial permeability transition pore (mPTP) on the inner mitochondrial membrane regulated by cyclophilin D (CypD) and/or increased outer mitochondrial membrane permeability mediated by Bax and Bak^7, 8^. Ferroptosis has recently been identified as a new form of regulated necrosis, which is characterized by iron-dependent lipid peroxidation, irreparable lipid damage, membrane disruption, and necrotic cell death^9, 10^. Glutathione peroxidase 4 (GPX4) is a key suppressor of ferroptosis, which is a glutathione (GSH)-dependent antioxidant enzyme that converts toxic lipid hydroperoxides to nontoxic lipid alcohols^11, 12^. GSH depletion or GPX4 inactivation has been linked to lipid ROS accumulation and ferroptotic cell death. Iron overload is a defining feature of ferroptosis, which promotes lipid ROS accumulation by producing hydroxyl and alkoxyl radicals through the Fenton reaction^13^. Notably, ferroptotic cell death, regardless the mechanisms of activation, can be blocked by iron chelators, indicating a central role of iron in the regulation and execution of ferroptosis. Moreover, cellular susceptibility to ferroptosis is closely linked to iron metabolism, including its import, export, utilization, and storage^9, 10, 14^. However, the mechanisms of iron overload during ferroptosis remain elusive, although several pathways have been proposed^15^.

Oxidative stress arising from excessive ROS production has been identified as either a trigger or a mediator of cell death. For example, it has been shown that oxidative stress is a major contributor of cardiomyocyte death in ischemia/reperfusion (I/R) injury as well as doxorubicin-induced cardiomyopathy^16–19^. Previous studies reported that cardiomyocytes undergo apoptosis when exposed to H_2_O_2_, an inorganic oxidant^20, 21^. Recent studies indicate that oxidative stress primarily induces necrosis, but not apoptosis, and the underlying mechanisms remain unclear. For example, Baines et al. reported that H_2_O_2_ induced mitochondria-dependent necrosis given that cells lacking CypD, a key component of mPTP, were resistant to H_2_O_2_ -induced cell death^7^. In contrast, Casey et al. showed that H_2_O_2_ induced a delayed form of necrosis which involves neither mPTP opening nor ATP depletion^22^. Moreover, H_2_O_2_ has been shown to induce RIPK1-dependent necroptosis in MEFs^23^, but other studies indicated that RIPK1 and RIPK3 were dispensable^24^. Similarly, controversial results have also been reported when organic oxidants, such as tert-butyl hydroperoxide (tBHP), are used to induce oxidative stress^25–27^. Therefore, the role of oxidative stress in cell death has not been unequivocally established, and the mechanisms by which oxidative stress induces distinct cell death signaling pathways are also elusive.

In this study, we sought to investigate the molecular mechanisms underlying oxidative stress-induced cell death in cardiomyocytes in vitro. We demonstrate for the first time that oxidative stress induced by organic oxidants primarily triggers ferroptosis, but not apoptosis, necroptosis, or mitochondria-mediated necrosis, in cardiomyocytes. We also delineated the molecular mechanisms underlying oxidative stress-induced cardiomyocyte ferroptosis by identifying signaling pathways driving lipid peroxidation and iron overload. Moreover, we identified mitochondrial iron overload via HO-1 mitochondrial translocation as a previously undescribed mechanism for oxidative stress-induced ferroptosis. Our results further suggest that targeting mitochondrial iron overload or lipid ROS accumulation may represent new cytoprotective strategies for oxidative stress-induced ferroptosis in cardiomyocytes.

## Materials and methods

### Reagents

tert-Butyl hydroperoxide, cumene hydroperoxide, hydrogen peroxide, ferrostatin-1, cyclosporine A, GSH, GSSG, and MG132 were from Sigma. Doxorubicin hydrochloride, liproxstatin-1, deferoxamine, mitoQ, and SKQ1 were from Cayman Chemical. MitoPeDPP and mito-FerroGreen were from Dojindo. Necrostatin-1s was from Cell Signaling Biotechnology. MitoSOX, propidium iodide, and Hoechst 33,342 were from Invitrogen. The following antibodies were used: anti-HMGB1 (3935), anti-HO-1 (82,206), anti-VDAC (4661), anti-catalase (14,097), and anti-GAPDH (2118) from Cell Signaling Biotechnology; anti-Bach1 (sc-271211) from Santa Cruz Biotechnology; Anti-FTMT (PAD251Mu01) from Cloud-Clone Corp.; anti-GPX4 from R&D Systems.

### Cell culture

All experiments involving animals were approved by the Institutional Animal Care and Use Committees of the University of Washington, and all studies were performed in accordance with relevant guidelines and regulations. All methods were reported in accordance with ARRIVE guidelines. Neonatal rat cardiomyocytes were isolated from hearts of 1- to 2-day-old Sprague–Dawley rat pups as previously described^28^. Briefly, neonatal hearts were collected, the atria were removed, and the ventricles were minced in HBSS prior to enzymatic digestion. The ventricular tissue was subjected to 5 rounds of enzymatic digestion using 0.05% pancreatin (Sigma-Aldrich) and 84 U/ml collagenase (Worthington). Cells were collected by centrifugation at 500*g* for 5 min at 4 °C and resuspended in M199 medium. After separation from fibroblasts, enriched cardiomyocytes were plated on culture dishes coated with 1% gelatin. Cells were grown in M199 medium supplemented with 2% bovine growth serum (Thermo Fisher Scientific), 100 U/ml penicillin–streptomycin, and 2 mM L-glutamine. For simulated ischemia/reperfusion (sI/R), ischemia was imposed by a buffer exchange to ischemic solution (20 mM HEPES, pH 6.6, 20 mM deoxyglucose, 125 mM NaCl, 8 mM KCl, 1.2 mM KH_2_PO_4_, 1.25 mM MgSO_4_, 1.2 mM CaCl_2_, 6.25 mM NaHCO_3_, 5 mM sodium lactate) and placing in a humidified chamber equilibrated with 95% N2 and 5% CO2. After 6 h of simulated ischemia, reperfusion was initiated by buffer exchange to normal culture medium in 95% room air and 5% CO2 for 12 h.

### Adenoviral vectors

Adenoviral vectors encoding GPX4 shRNA, HO-1 shRNA, Bach1 shRNA, and FTMT shRNA were generated using the BLOCK-iT Adenoviral RNAi Expression System (Invitrogen) according to the manufacturer’s instructions. The core sequence for GPX4 shRNA: 5′-GCCAGGAAGTAATCAAGAAAT-3′; HO-1 shRNA: 5′-GCTGACAGAGGAACACAAAGA-3′; Bach1 shRNA: 5′- GCGTACACAATATCGAGGAAT-3′; FTMT shRNA: 5′-GCTTTACGCATCCTACGTGTA-3′. To generate adenoviral vectors for HO-1 and FTMT, HO-1-2A-EGFP (Addgene #74672) and FTMT-Flag (GenScript #OHu55907) were cloned into pAd/CMV/V5-DEST using the ViraPower Adenoviral Expression System (Invitrogen). Ad-GPX4 was obtained from ViraQuest Inc. Ad-mCAT was obtained from University of Iowa Vector Core. Adenoviral infections were performed at a multiplicity of infection of 10 to 50 plaque forming units per ml^29^.

### Cell death assays

Cell death was assessed using a Cell Meter Apoptotic and Necrotic Detection kit (ATT Bioquest) as we previously described^30^. Briefly, cells were incubated at 37 °C for 30 min with Apopxin Green for detection of phosphatidylserine on cell surface, propidium iodide (PI) or 7-ADD for labeling the nucleus of cells with membrane rupture, and CytoCalcein for labeling live cell cytoplasm. Cell death was analyzed with an EVOS FL digital fluorescence microscope (AMG) and the Muse cell analyzer (Millipore). All imaging data are representative of at least three randomly selected fields.

### Western blotting

Cell culture supernatants were collected and centrifuged at 500*g* for 10 min at 4 °C to remove detached cells. Cells were lysed using RIPA buffer (50 mM Tris pH 7.5, 150 mM NaCl, 1% NP-40, 0.5% sodium deoxycholate, and 0.1% sodium dodecyl sulfate, 2 mM DTT, 2 mM sodium orthovanadate, 1 × protease inhibitor cocktail [Roche]). Equal amounts of protein were subjected to SDS-PAGE and transferred to PVDF membranes (Millipore). Western blotting followed by enhanced chemiluminescence detection was performed as previously described^30^.

### Glutathione measurement

Glutathione levels were measured using a glutathione assay kit (Cayman Chemical) according to the manufacturer's instructions. Briefly, cells were re-suspended in MES buffer (0.4 M 2-ethanesulphonic acid, 0.1 M phosphate, 2 mM EDTA, pH 6.0) and lysed by sonification. After centrifugation at 10,000*g* for 15 min, 1.25 M metaphosphoric acid was added to the supernatant for precipitation of proteins, followed by centrifugation at 15,000*g* for 10 min. The supernatant was transferred into a 96-well plate and incubated with the assay cocktail containing MES-buffer, co-factor and enzyme mixture, and Ellman's reagent for 30 min. Absorbance was measured at 405 nm with a BioTek Synergy 2 microplate reader (BioTek).

### Measurement of lipid peroxidation

Lipid peroxidation was measured using C11-BODIPY 581/591 (Invitrogen) according to the manufacturer's instructions. Cells were incubated in 10 μM C11-BODIPY581/591 for 30 min at 37 °C. Fluorescence measurements were performed using a BioTek Synergy 2 microplate reader with excitation wavelength of 581 nm and an emission wavelength of 591 nm.

### Labile iron levels

The labile iron levels in cardiomyocytes were measured using the calcein-AM method (Yoshida M 3145). Briefly, cells were incubated with 1 μM calcein-AM at 37 °C for 10 min followed by washing with PBS. Fluorescence intensity was measured using a BioTek Synergy 2 fluorescence microplate reader. Cells were then treated with 100 μM 2′,2′-bipyridine (BIP) at 37 °C for 10 min and the fluorescence was measured again. The changes in fluorescence upon BIP treatment was used to determine the labile iron pool.

### Cytosolic and mitochondrial fractions

Cytosolic and mitochondrial fractions were prepared based on the method by Frezza et al. with some modifications^31^. Briefly, cells were suspended in sucrose-mannitol buffer (20 mM HEPES, pH 7.5, 2 mM EDTA, 70 mM sucrose, 220 mM mannitol, 5 mM NaF, protease inhibitor cocktail [Roche]) and homogenized using a Teflon homogenizer. The homogenates were centrifuged at 600*g* for 10 min at 4 °C. The supernatant was re-centrifuged at 10,000×*g* for 15 min at 4 °C to collect the supernatant (cytosolic fraction) and pellet (mitochondrion fraction). The purity of cytosolic and mitochondrial fractions was validated by Western blotting using anti-GAPDH and anti-VDAC antibodies, respectively.

### Mitochondrial iron

Mitochondrial iron was measured using mito-FerroGreen (Dojindo), a fluorescence probe for mitochondrial ferrous ion (Fe^2+^). Cells were incubated in 5 μM mito-FerroGreen for 30 min at 37 °C. After three washes in PBS, 100 μM ammonium iron sulfate was then added to the cells. Mitochondrial iron was fluorometrically measured using a BioTek Synergy 2 microplate reader at an excitation wavelength of 505 nm and an emission wavelength of 535 nm or visualized using an EVOS FL fluorescence microscope.

### Mitochondrial lipid peroxidation

Mitochondrial lipid peroxidation was assessed using mitoPeDPP (Dojindo), a fluorescence probe that specifically detects lipid peroxides in the mitochondrial inner membrane. Cells were incubated with 0.5 μM mitoPeDPP solution for 30 min at 37 °C. After three washes with PBS, mitochondrial lipid peroxidation was fluorometrically measured using a BioTek Synergy 2 microplate reader at an excitation wavelength of 452 nm and an emission wavelength of 470 nm.

### Measurement of mitochondrial ROS

MitoSOX Red (Invitrogen) was used for analyzing mitochondrial ROS. Cells were loaded with MitoSOX at 5 µM concentration for 30 min at 37 °C. After washing three times with PBS, fluorescence was detected by an EVOS FL digital fluorescence microscope (AMG) and quantified using ImageJ software. Data were collected from at least 3000 cells.

### Statistics

Results are presented as mean ± SEM. Statistical analysis was performed using GraphPad Prism 9 (GraphPad). Statistical analysis was performed using the Student’s two-tailed t test for comparison between 2 groups. Comparisons between multiple groups were made using one-way analysis of variance (ANOVA) with Tukey’s post hoc test. Comparison of multiple groups with multiple conditions was performed using 2-way ANOVA with Tukey’s multiple-comparison test. *P* < 0.05 was considered significant.

## Results

### Organic oxidants induce ferroptosis in cardiomyocytes

**Figure 1:**
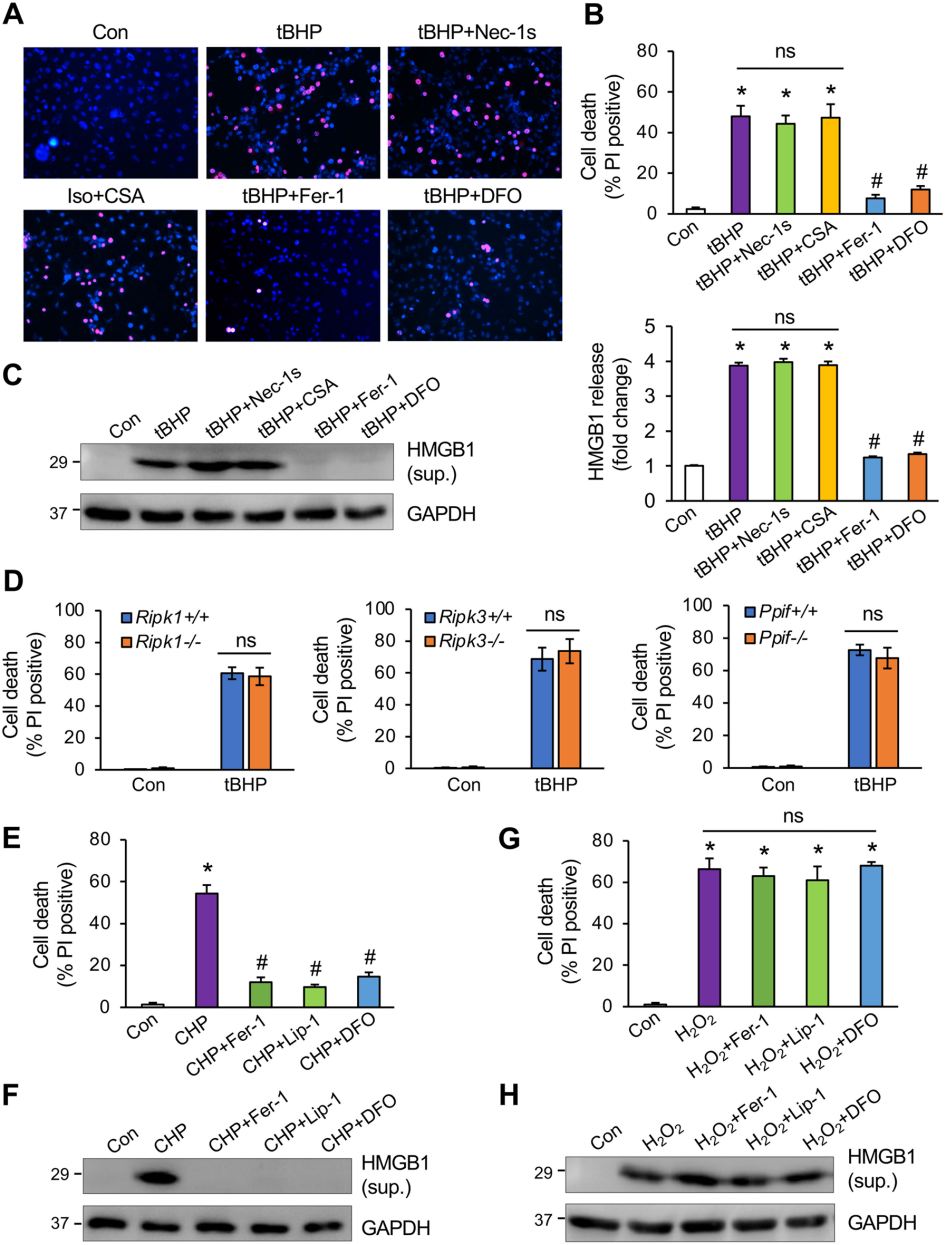
Organic oxidants induce ferroptotic cell death in cardiomyocytes. (**A**) Cell death assessed by propidium iodide (red) and Hoechst 33,342 (blue) staining of cardiomyocytes treated with vehicle control or 100 μM tBHP, with or without 1 μM necrostatin-1s (Nec-1s), 1 μM Cyclosporin A (CsA), 10 μM ferrostatin-1 (Fer-1), or 10 μM deferoxamine (DFO) for 12 h. (**B**) Quantification of cell death from cells treated as in A. *P < 0.001 vs Control; #*P* < 0.05 vs tBHP. n = 3 independent experiments. (**C**) Western blotting for HMGB1 in the culture supernatant (sup.) and GAPDH in whole cell extracts from cells treated as in A. HMGB1 levels were quantified as fold changes over control. *P < 0.001 vs Control; #*P* < 0.05 vs tBHP. n = 3. (**D**) Quantification of cell death from the indicated MEF cell lines treated with tBHP or vehicle control for 12 h. ns, non-significant. n = 3. (**E**) Cell death from cardiomyocytes treated with vehicle control or 100 μM CHP, with or without 10 μM Fer-1, 1 μM liproxstatin-1 (Lip-1), or 10 μM DFO for 12 h. *P < 0.001 vs Control; #*P* < 0.05 vs CHP. n = 3. (**F**) Western blotting for HMGB1 in the culture supernatant (sup.) and GAPDH in whole cell extracts from cells treated as in E. (**G**) Cell death from cardiomyocytes treated with vehicle control or 200 μM H_2_O_2_, with or without Fer-1, Lip-1, or DFO for 12 h. *P < 0.001 vs Control. ns, non-significant. n = 3. (**H**) Western blotting for HMGB1 in the culture supernatant (sup.) and GAPDH in whole cell extracts from cells treated as in G.

Oxidative stress primarily induces non-apoptotic cell death, but the cell death mechanism remains unclear^7, 22^. Here, we examined whether and how oxidative stress induces ferroptosis in cardiomyocytes. tert-butyl hydroperoxide (tBHP), an organic peroxide, was used to generate oxidative stress, which induced necrotic cell death in cardiomyocytes, as indicated by increased propidium iodide (PI) uptake, an indicator of impaired plasma membrane integrity (Fig. 1A,B). Moreover, tBHP also promoted the release of high mobility group box 1 (HMGB1) into the culture supernatant, another marker of necrotic cell death^30^ (Fig. 1C). Importantly, tBHP-induced cell death and HMGB1 release were largely inhibited by classic ferroptosis inhibitors such as ferrostatin-1 (Fer-1) and deferoxamine (DFO), indicating the induction of ferroptosis (Fig. 1A-C). In contrast, tBHP-induced cell death was not affected by necrostatin-1 s (Nec-1 s) or cyclosporine A (CsA), suggesting a mechanism distinct from necroptosis or mitochondria-mediated necrosis (Fig. 1A-C). Consistent with this observation, genetic deletion of *Ripk1*, *Ripk3,* or *Ppif* (CyPD) also had minimal effects on tBHP-induced cell death in mouse embryonic fibroblasts (MEFs) (Fig. 1D). These results indicate that tBHP primarily induces ferroptosis, but not necroptosis or mitochondria-dependent necrosis, in cardiomyocytes.

The effect of oxidative stress-induced cell death was further assessed using cumene hydroperoxide (CHP) and H_2_O_2_ as the oxidizing agents. Like tBHP, CHP markedly induced cell death and HMGB1 release in cardiomyocytes, which was effectively inhibited by ferroptosis inhibitors such as Fer-1, DFO, and liproxstatin-1 (Lip-1) (Fig. 1E,F). Intriguingly, H_2_O_2_ also induced cardiomyocyte death and HMGB1 release, but these effects were resistant to ferroptosis inhibition (Fig. 1G,H). These results reveal that ferroptosis is selectively induced by organic oxidants but not H_2_O_2_.

### Organic oxidants promote glutathione depletion, GPX4 downregulation, and enhanced lipid peroxidation in cardiomyocytes

**Figure 2:**
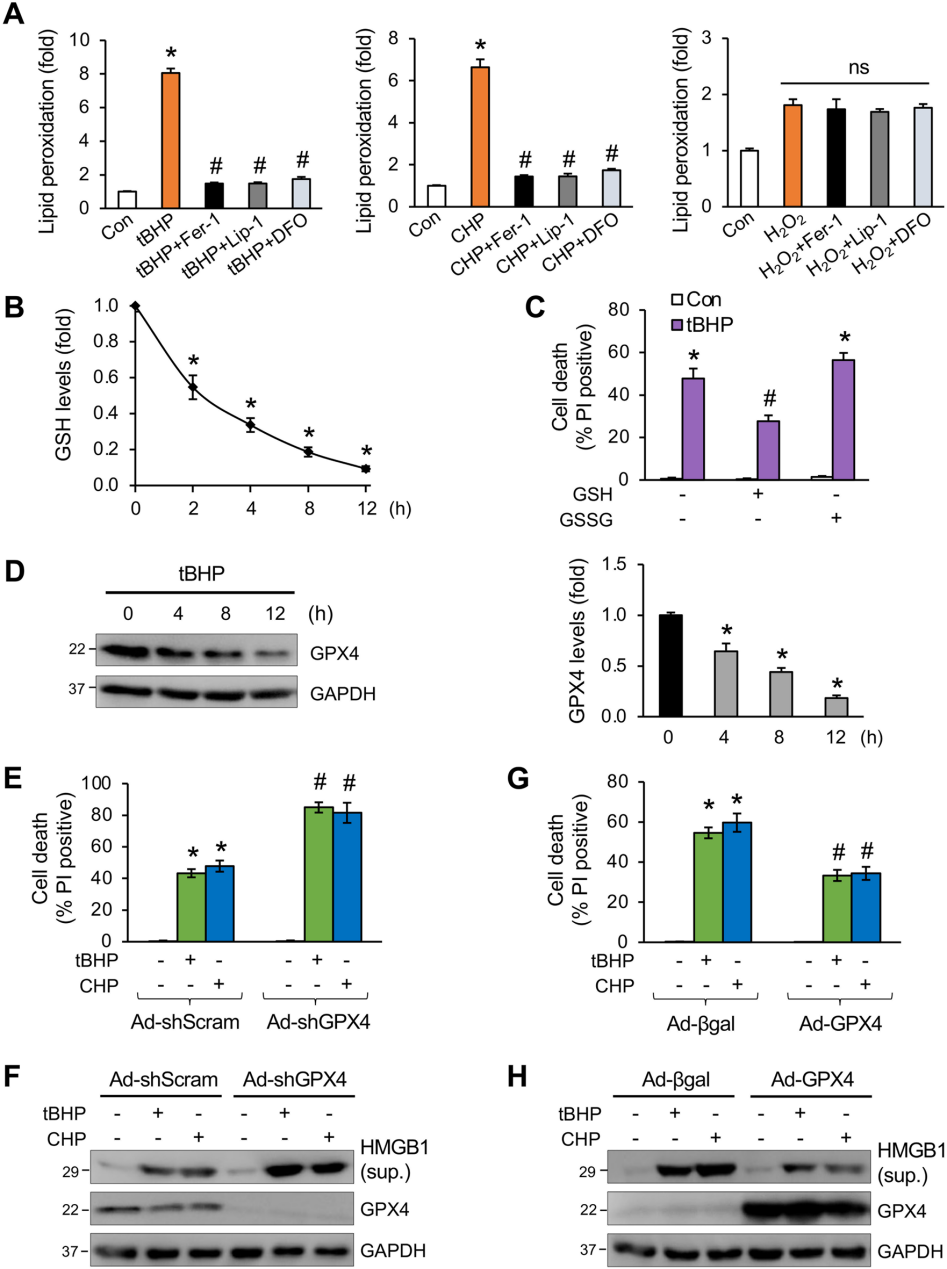
Organic oxidants promote glutathione depletion, GPX4 downregulation, and lipid peroxidation in cardiomyocytes. (**A**) Quantification of lipid peroxidation using C11-BODIPY 581/591 in cardiomyocytes treated with vehicle control, tBHP, CHP, or H_2_O_2_, with or without Fer-1, Lip-1, or DFO for 8 h. *P < 0.001 vs Control; #*P* < 0.05 vs tBHP or CHP. n = 3. (**B**) GSH levels in cardiomyocytes treated with tBHP for the indicated time periods. *P < 0.05 vs 0 h. n = 4. (**C**) Cell death from cardiomyocytes pretreated with 2 mM GSH or GSSG for 30 min followed by tBHP for 12 h. *P < 0.001 vs Control. *P < 0.05 vs tBHP only. n = 3. (**D**) Western blotting and quantification for GPX4 expression in cardiomyocytes treated with tBHP for the indicated time periods. *P < 0.05 vs 0 h. n = 3. (**E**) Quantification of cell death from cardiomyocytes infected with adenoviral vectors encoding GPX4-shRNA (Ad-shGPX4) or a scrambled sequence (Ad-shScram) for 24 h followed by treatment with tBHP, CHP, or vehicle control for 12 h. *P < 0.001 vs Control; #*P* < 0.05 vs Ad-shScram + tBHP or CHP. n = 3. (**F**) Western blotting for HMGB1 in the culture supernatant (sup.) and GAPDH in whole cell extracts from cells treated as in E. (**G**) Quantification of cell death from cardiomyocytes infected with adenoviral vectors encoding GPX4 (Ad-GPX4) or β-galactosidase (Ad-βgal) for 24 h followed by treatment with tBHP, CHP, or vehicle control for 12 h. *P < 0.001 vs Control; #*P* < 0.05 vs Ad-βgal + tBHP or CHP. n = 3. (**H**) Western blotting for HMGB1 in the culture supernatant (sup.) and GAPDH in whole cell extracts from cells treated as in G.

Here, we examined whether organic oxidants promote lipid peroxidation, a key feature of ferroptosis. Indeed, both tBHP and CHP greatly induced lipid peroxidation in cardiomyocytes, which was largely blocked by Fer-1, Lip-1, or DFO (Fig. 2A). In contrast, H_2_O_2_ only moderately increased lipid peroxidation, which was not affected by treatment with Fer-1, Lip-1, or DFO (Fig. 2A). These results are consistent with our finding that tBHP and CHP, but not H_2_O_2_, induced ferroptosis in cardiomyocytes.

To understand the mechanism of organic oxidants -induced ferroptosis, we assessed the effect of tBHP on GSH-GPX4 signaling. We found that tBHP induced GSH depletion in cardiomyocytes in a time-dependent manner (Fig. 2B). Pretreatment with the reduced GSH, but not the oxidized GSSG, inhibited tBHP-induced cell death (Fig. 2C). Moreover, GPX4 was also markedly downregulated upon tBHP stimulation (Fig. 2D). To further determine the role of GPX4 in tBHP-induced ferroptosis, we examined the rates of cell death in cardiomyocytes transduced with adenoviral vectors encoding GPX4 shRNA or wild-type GPX4. GPX4 silencing further promoted tBHP-induced cell death and HMGB1 release, whereas GPX4 overexpression showed the opposite effects (Fig. 2E-H), suggesting that GPX4 confers cell death resistance to oxidative stress. These results indicate that organic oxidants induce cardiomyocyte ferroptosis through GSH depletion and GPX4 downregulation.

### Organic oxidants promote labile iron accumulation via the Bach1-HO-1 pathway

**Figure 3:**
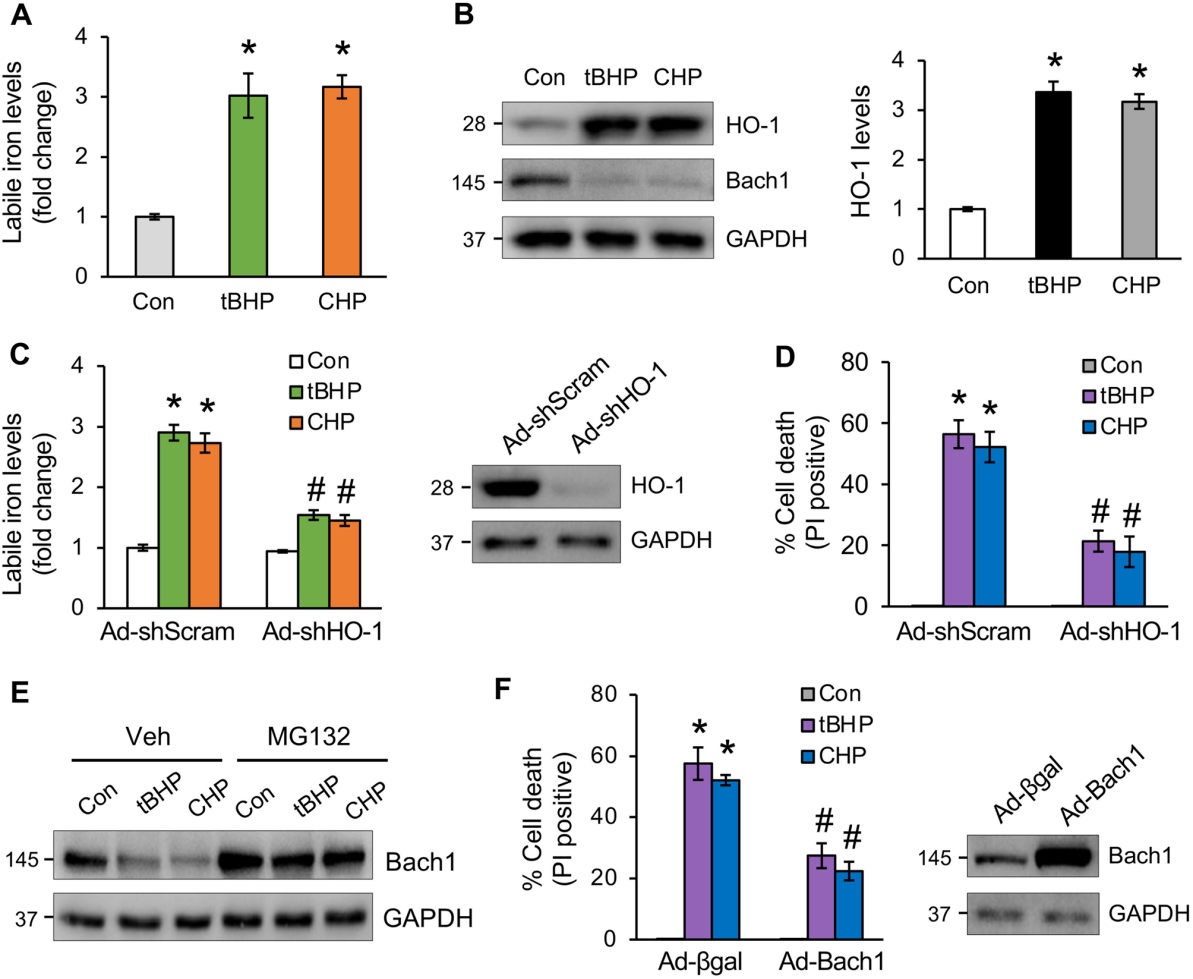
Organic oxidants promote labile iron accumulation via the Bach1-HO-1 pathway. (**A**) Assessment of labile iron levels in cardiomyocytes treated with tBHP, CHP, or vehicle control for 8 h. *P < 0.05 vs Control. n = 3. (**B**) Western blotting and quantification for HO-1 expression in cells treated as in A. *P < 0.05 vs Control. n = 3. (**C**) Labile iron levels in cardiomyocytes infected with adenoviral vectors encoding HO-1 shRNA (Ad-shHO-1) or Ad-shScram for 24 h followed by tBHP, CHP, or vehicle control for 8 h. *P < 0.05 vs Control; #*P* < 0.05 vs Ad-shScram + tBHP or CHP. n = 4. (**D**) Quantification of cell death from cells treated as in C for 12 h. *P < 0.05 vs Control; #*P* < 0.05 vs Ad-shScram + tBHP or CHP. n = 3. (**E**) Western blotting for Bach1 in cardiomyocytes pretreated with MG132 or vehicle control for 30 min followed by tBHP or CHP for 4 h. (**F**) Quantification of cell death from cardiomyocytes infected with adenoviral vectors encoding Bach1 (Ad-Bach1) or Ad-βgal for 24 h followed by treatment with tBHP, CHP, or vehicle control for 12 h. *P < 0.001 vs Control; #*P* < 0.05 vs Ad-βgal + tBHP or CHP. n = 4.

Next, we examined whether oxidative stress induces iron overload, which is a defining feature of ferroptosis. Indeed, both tBHP and CHP greatly increased labile iron levels in cardiomyocytes as assessed by calcein-acetoxymethyl ester assay^32^ (Fig. 3A). Moreover, tBHP-induced iron overload correlates with a marked upregulation of heme oxygenase-1 (HO-1), an enzyme that drives iron release via heme degradation^33^. Conversely, the transcription factor BTB and CNC homology 1 (Bach1), a transcriptional suppressor for HO-1^34^, was downregulated by tBHP and CHP (Fig. 3B). Deletion of HO-1 markedly reduced labile iron levels induced by tBHP or CHP, further confirming the role of HO-1 in oxidative stress-induced iron overload (Fig. 3C). Moreover, deletion of HO-1 also inhibited tBHP- or CHP-induced cardiomyocyte death (Fig. 3D). Similar effect was obtained in cardiomyocytes treated with zinc protoporphyrin IX (ZnPP), an HO-1 inhibitor (Supplemental Fig. 1). Conversely, HO-1 overexpression further promoted tBHP-induced cell death (Supplemental Fig. 2A). To determine the mechanism by which oxidative stress promotes HO-1 expression, we showed that tBHP or CHP promoted the degradation of Bach1, which was blocked by pretreatment with the proteasome inhibitor MG-132 (Fig. 3E). Moreover, deletion of Bach1 with an adenoviral vector encoding Bach1 shRNA was sufficient to promote HO-1 expression in cardiomyocytes (Supplemental Fig. 2B), suggesting that oxidative stress induces HO-1 expression through Bach1 degradation. Moreover, overexpression of Bach1 inhibited cell death tBHP- or CHP-induced cell death (Fig. 3F). Together, these results reveal a key role for the Bach1-HO-1 signaling pathway in organic oxidants-induced iron overload and ferroptosis in cardiomyocytes.

### Organic oxidants promote mitochondrial iron overload

**Figure 4:**
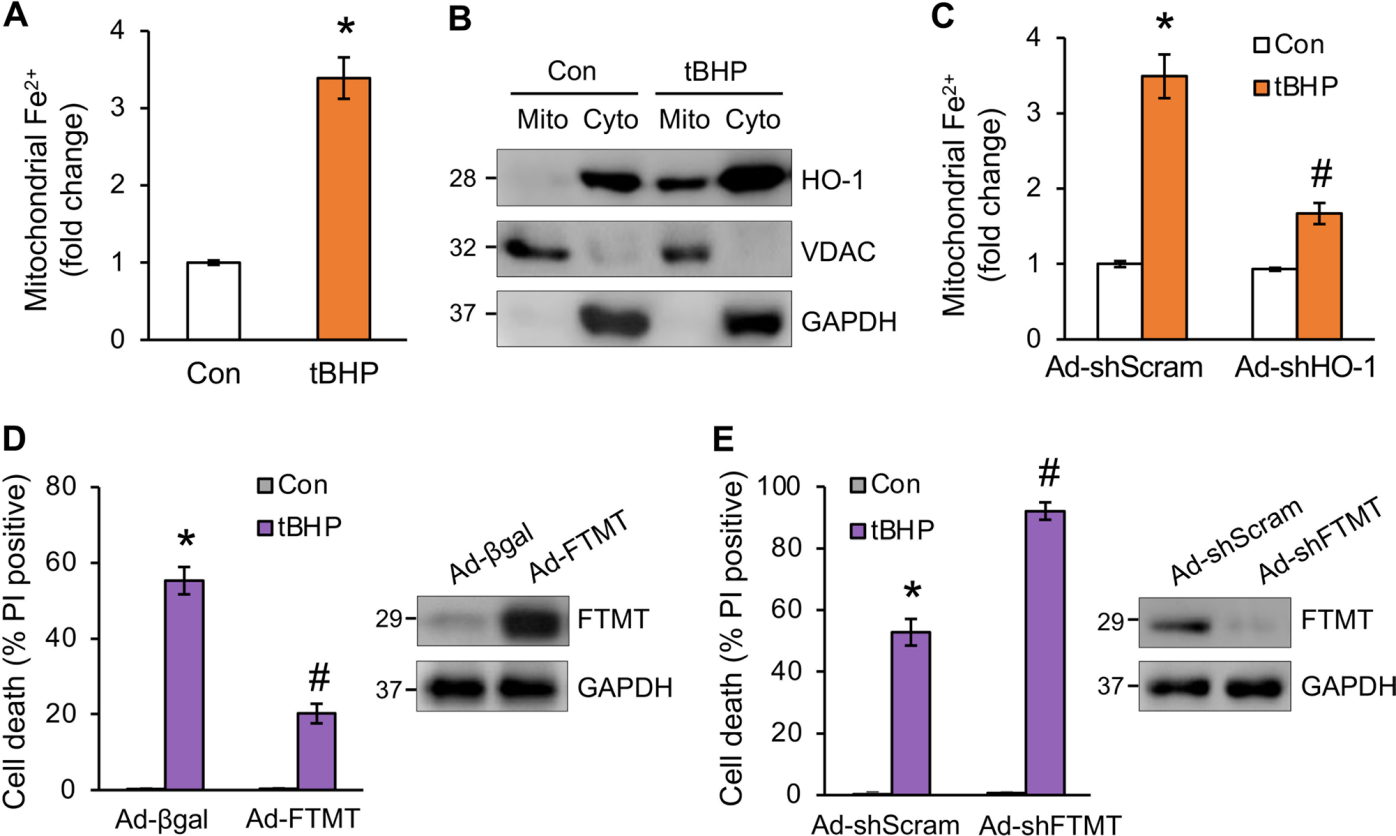
Organic oxidants promote mitochondrial iron overload. (**A**) Mitochondrial Fe^2+^ levels assessed by mito-FerroGreen in cardiomyocytes treated with tBHP or vehicle control for 8 h. *P < 0.05 vs Control. n = 4. (**B**) Western blotting for the indicated proteins from mitochondrial and cytosolic fractions of cardiomyocytes treated with tBHP or vehicle control. (**C**) Mitochondrial Fe^2+^ levels in cardiomyocytes infected with Ad-shHO-1 or Ad-shScram for 24 h followed by tBHP treatment for 8 h. *P < 0.05 vs Control; #*P* < 0.05 vs Ad-shScram + tBHP. n = 4. (**D**) Quantification of cell death from cardiomyocytes infected with adenoviral vectors encoding mitochondrial ferritin (Ad-FTMT) or Ad-βgal for 24 h followed by tBHP treatment for 12 h. FTMT expression was assessed by Western blotting. *P < 0.001 vs Control; #*P* < 0.05 vs Ad-βgal + tBHP. n = 4. (**E**) Quantification of cell death from cardiomyocytes infected with adenoviral vectors encoding FTMT-shRNA (Ad-shFTMT) or Ad-shScram for 24 h followed by tBHP treatment for 12 h. *P < 0.001 vs Control; #*P* < 0.05 vs Ad-shScram + tBHP. n = 3.

The role of mitochondria in ferroptosis has been controversial, possibly depending on cell types and cellular contexts^35–38^. Here, we identified a critical role of mitochondria in mediating oxidative stress-induced ferroptosis in cardiomyocytes. Intriguingly, a marked increase in mitochondrial ferrous iron (Fe^2+^) was detected in cardiomyocytes after tBHP treatment (Fig. 4A). Strikingly, this effect was associated with a significant translocation of HO-1 from the cytosol to mitochondria (Fig. 4B). Moreover, tBHP induced mitochondrial iron accumulation was largely abrogated by HO-1 deletion, revealing a key role for HO-1 in mediating mitochondrial iron overload (Fig. 4C). Importantly, overexpressing mitochondrial ferritin (FTMT), a mitochondrial matrix protein that chelates iron, effectively inhibited tBHP-induced cell death in cardiomyocytes (Fig. 4D), further suggesting that mitochondrial iron overload plays a key role in tBHP-induced ferroptosis. Conversely, deletion of FTMT further promoted tBHP-induced cell death in cardiomyocytes (Fig. 4E). In contrast to FTMT, overexpression of ferritin heavy chain 1 (FTH1), which chelates cytosolic iron, moderately inhibited ferroptosis (Supplemental Fig. 3). Together, these data identified mitochondrial iron overload as a key mediator of organic oxidants-induced ferroptosis in cardiomyocytes.

### Organic oxidants promote mitochondrial lipid peroxidation and ROS accumulation

**Figure 5:**
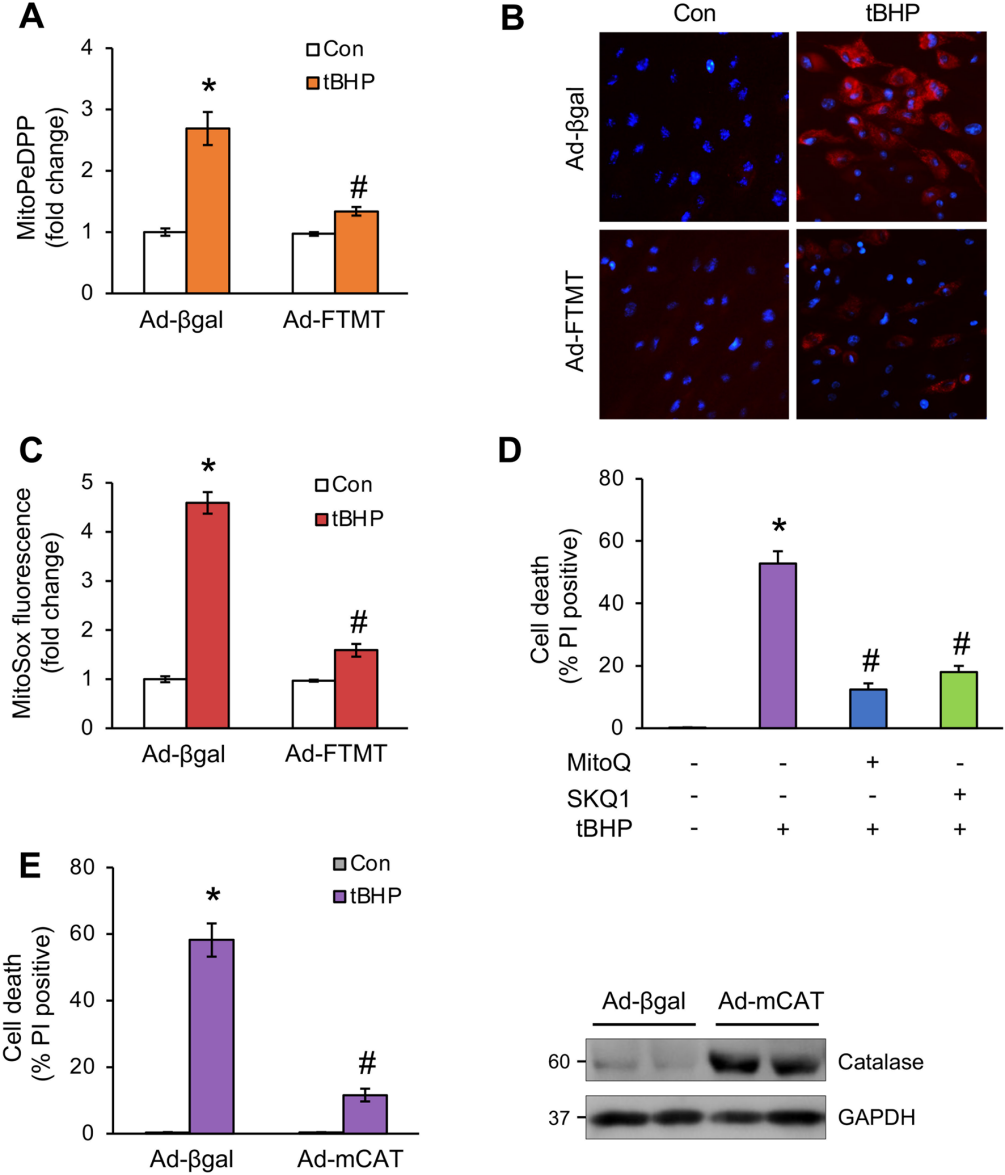
Organic oxidants promote mitochondrial lipid peroxidation and ROS accumulation. (**A**) Mitochondrial lipid peroxidation assessed with MitoPeDPP in cardiomyocytes infected with Ad-FTMT or Ad-βgal for 24 h followed by tBHP treatment for 8 h. *P < 0.05 vs Control; #*P* < 0.05 vs Ad-βgal + tBHP. n = 4. (**B**) Representative fluorescence images of MitoSox staining from cardiomyocytes treated as in A. (**C**) Quantification of MitoSox fluorescence from cells treated as in A. *P < 0.001 vs Control; #*P* < 0.05 vs Ad-βgal + tBHP. n = 4. (**D**) Quantification of cell death from cardiomyocytes treated with tBHP or vehicle in the presence or absence of mitoquinol (MitoQ) or SKQ1 for 12 h. *P < 0.001 vs Control; #*P* < 0.05 vs tBHP only. n = 3. (**E**) Cell death was assessed from cardiomyocytes infected with adenoviral vectors for mitochondria-targeted catalase (mCAT) or Ad-βgal for 24 h followed by tBHP treatment for 12 h. Catalase expression was assessed by Western blotting. *P < 0.001 vs Control; #*P* < 0.05 vs Ad-βgal + tBHP. n = 3.

tBHP also induced lipid peroxidation in the mitochondrial fraction as assessed by a mitochondria-targeted fluorescent sensor mitoPeDPP (Fig. 5A). This effect was largely inhibited by FTMT overexpression, revealing a link between iron overload and lipid peroxidation within the mitochondria (Fig. 5A). In contrast to tBHP, H_2_O_2_ had no significant effects on mitochondrial lipid peroxidation (Supplemental Fig. 4). In addition, H_2_O_2_ only mildly reduced cellular GSH levels (Supplemental Fig. 4). The levels of mitochondrial ROS, as measured by mitoSOX, were also greatly elevated upon tBHP stimulation, which was also abrogated by FTMT overexpression (Fig. 5B,C). Moreover, pretreatment with mitochondria-targeted ROS scavengers^39^, such as mitoquinol (MitoQ) and SKQ1, also inhibited tBHP-induced cell death (Fig. 5D). Overexpressing mitochondria-targeted catalase (mCAT) also effectively inhibited tBHP-induced cell death (Fig. 5E). Together, these results suggest that mitochondrial iron overload and lipid peroxidation critically mediate organic oxidants-induced cardiomyocyte ferroptosis.

### Mitochondrial iron and ROS accumulation mediates cardiomyocyte death induced by simulated ischemia/reperfusion or doxorubicin

**Figure 6:**
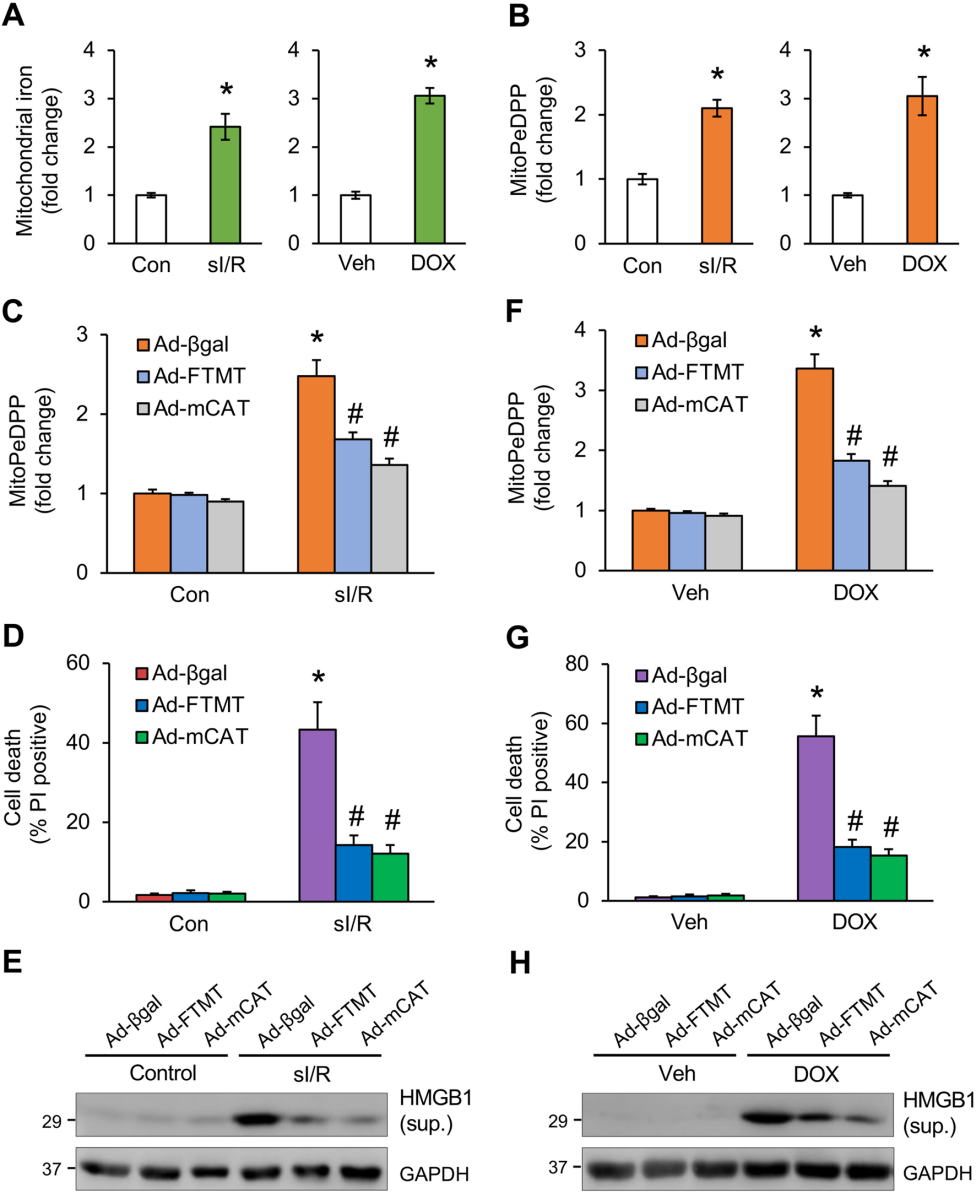
Inhibition of mitochondrial iron or ROS accumulation prevented simulated ischemia/reperfusion or doxorubicin induced cell death in cardiomyocytes. (**A**) Mitochondrial Fe^2+^ levels in cardiomyocytes subjected to 6 h simulated ischemia and 12 h reperfusion (sI/R) or control condition (left panel). Mitochondrial Fe^2+^ levels were also measured in cells treated with 10 μM DOX or vehicle control for 12 h (right panel). *P < 0.05 vs Control or Veh. n = 4. (**B**) Mitochondrial lipid peroxidation assessed with MitoPeDPP in cardiomyocytes subjected to sI/R or DOX as in A. *P < 0.05 vs Control or Veh. n = 4. (**C**) Mitochondrial lipid peroxidation assessed with MitoPeDPP in cardiomyocytes infected with Ad-βgal, Ad-FTMT, or Ad-mCAT and subjected to sI/R or control condition. *P < 0.05 vs Control. #*P* < 0.05 vs Ad-βgal sI/R. n = 3. (**D**) Quantification of cell death from cardiomyocytes infected with Ad-βgal, Ad-FTMT, or Ad-mCAT and subjected to sI/R or control condition. *P < 0.05 vs Control. #*P* < 0.05 vs Ad-βgal sI/R. n = 4. (**E**) Western blotting for the indicated proteins from cells treated as in D. (**F**) Mitochondrial lipid peroxidation assessed with MitoPeDPP in cardiomyocytes infected with Ad-βgal, Ad-FTMT, or Ad-mCAT followed by treatment with DOX or vehicle control for 12 h. *P < 0.05 vs Veh. #*P* < 0.05 vs Ad-βgal DOX. n = 3. (**G**) Quantification of cell death from cardiomyocytes infected with Ad-βgal, Ad-FTMT, or Ad-mCAT followed by treatment with DOX or vehicle control for 12 h. *P < 0.05 vs Veh. #*P* < 0.05 vs Ad-βgal DOX. n = 4. (**H**) Western blotting for the indicated proteins from cells treated as in G.

To further investigate the role of mitochondrial iron and ROS in pathological conditions associated with elevated oxidative stress^17, 19^, cardiomyocytes were subjected to simulated ischemia and reperfusion (sI/R) or the chemotherapeutic agent doxorubicin (DOX). Mitochondrial iron levels were markedly elevated in cardiomyocytes subjected to sI/R or DOX (Fig. 6A). Elevated mitochondrial lipid peroxidation was also detected under these conditions (Fig. 6B). To examine the role of mitochondrial iron and ROS in sI/R- or DOX-induced cell death, cardiomyocytes were transduced with Ad-FTMT or Ad-mCAT to prevent mitochondrial iron or ROS accumulation. Overexpressing FTMT or mCAT diminished mitochondrial lipid peroxidation and cell death induced by sI/R, associated with reduced HMGB1 release (Fig. 6C-E). Similar effects were obtained in cardiomyocytes treated with DOX (Fig. 6F-H). These results suggest that targeted inhibition of mitochondrial iron or ROS accumulation prevents cell death in pathological conditions associated with oxidative stress.

## Discussion

**Figure 7:**
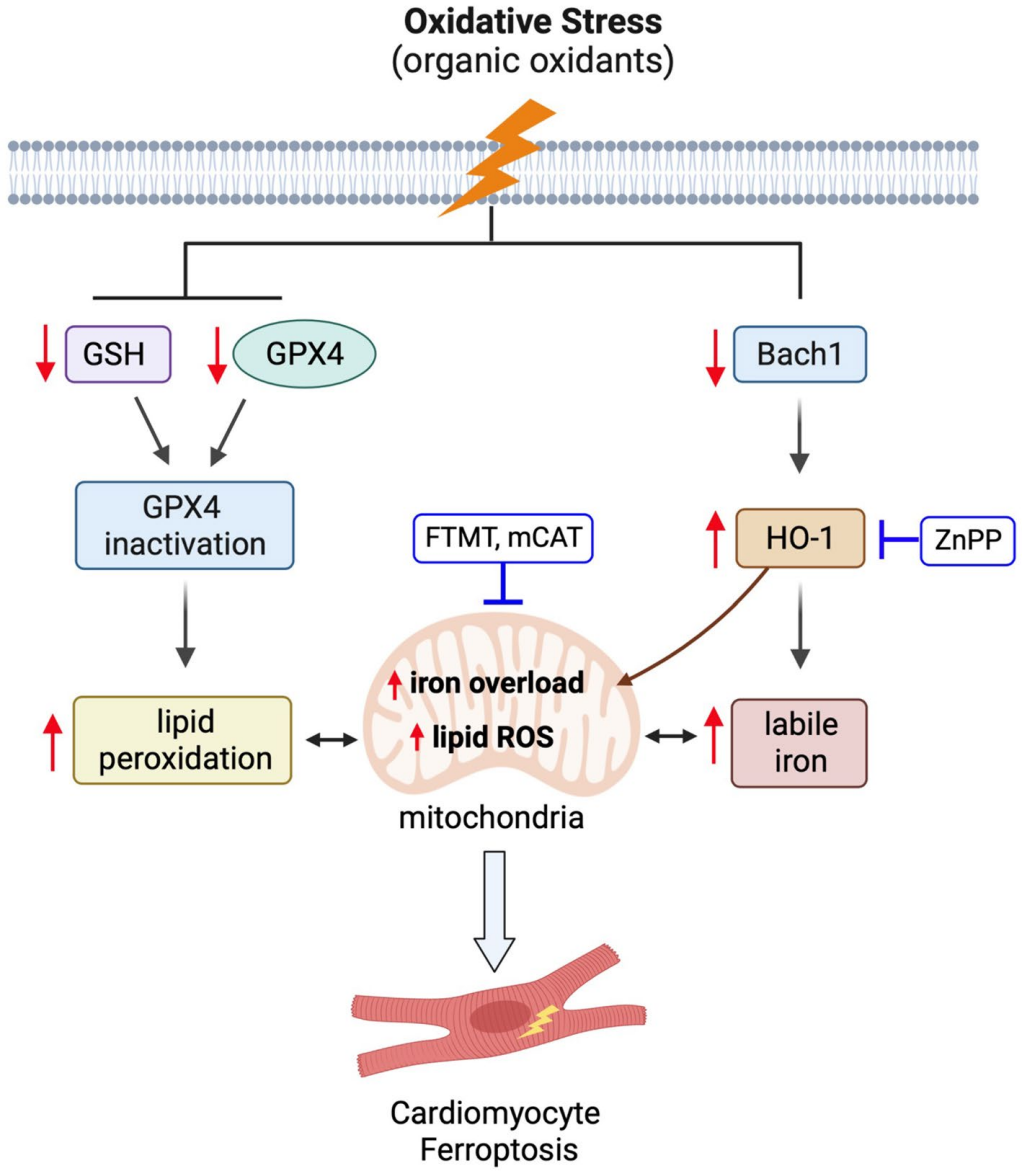
Proposed mechanisms by which oxidative stress induces mitochondrial iron overload and ferroptosis in cardiomyocytes. Oxidative stress induced by organic oxidants promotes GSH depletion and GPX4 downregulation, leading to increased lipid peroxidation. Oxidative stress also increases labile iron levels through downregulation of the transcription suppressor Bach1, upregulation of HO-1 expression, and enhanced iron release via heme degradation. Moreover, oxidative stress also promotes HO-1 translocation to mitochondria, leading to mitochondrial iron overload and lipid ROS accumulation. Targeted inhibition of mitochondrial iron overload and ROS accumulation prevents oxidative stress-induced ferroptosis in cardiomyocytes. ZnPP, zinc protoporphyrin IX, HO-1 inhibitor; FTMT, mitochondrial ferritin; mCAT, mitochondrial catalase.

In the present study, we provide new evidence that oxidative stress induced by organic oxidants primarily causes ferroptotic cell death in cardiomyocytes, highlighting the significance of this new cell death modality in heart disease driven by elevated oxidative stress. Mechanistically, oxidative stress promotes GSH depletion and GPX4 downregulation, leading to enhanced lipid peroxidation (Fig. 7). Moreover, we provide mechanistic evidence linking elevated oxidative stress to labile iron overload through the Bach1-HO-1 signaling (Fig. 7). Our data also reveal the interdependence of lipid peroxidation and iron overload in ferroptosis signaling, as blockade of either pathway prevented oxidative stress-induced ferroptosis in cardiomyocytes. Importantly, we further identified HO-1 mitochondrial translocation as a previously undescribed mechanism that mediates iron overload and lipid ROS accumulation within the mitochondria (Fig. 7). Strikingly, targeted inhibition of mitochondrial iron overload and ROS accumulation, by overexpressing FTMT or mCAT, respectively, markedly inhibited oxidative stress-induced ferroptosis. These results suggest that mitochondrial iron overload and lipid ROS accumulation may represent potential therapeutic targets in oxidative stress-induced pathological conditions.

Here we showed that organic oxidants such as tBHP and CHP, but not H_2_O_2_, primarily induced ferroptosis in cardiomyocytes. Consistent with this observation, tBHP and CHP greatly enhanced lipid peroxidation in cardiomyocytes, whereas H_2_O_2_ only had moderate effect. These results reveal distinct effects of different oxidative stress inducers, possibly depending on their chemical properties. Indeed, it has been shown that tBHP can be metabolized to produce peroxyl and alkoxyl radicals^40^, which can initiate lipid peroxidation of membrane phospholipids. Mechanistically, we further show that tBHP induces GSH depletion as well as GPX4 downregulation in cardiomyocytes, leading to GPX4 inactivation and elevated lipid peroxidation. Notably, GSH depletion and/or GPX4 downregulation have also been linked to pathological oxidative stress in vivo, such as ischemia/reperfusion cardiac injury and doxorubicin induced cardiomyopathy^41, 42^.

It is well established that iron overload promotes ROS accumulation via the Fenton reaction^43^, but whether the reverse is also true has not been directly investigated. Here we show that elevated oxidative stress can also induce iron overload in cardiomyocytes. Mechanistically, we found that oxidative stress promoted the expression of HO-1, an enzyme that drives labile iron release through heme degradation. Notably, ablation of HO-1 abolished, whereas overexpression of HO-1 exacerbated, oxidative stress-induced labile iron overload and ferroptosis. These data suggest that oxidative stress-induced iron overload is mainly originated intracellularly through HO-1 mediated iron release. It is possible that iron overload can also occur through additional mechanisms, such as nuclear receptor coactivator 4 (NCOA4) mediated ferritinophagy and lysosomal iron release^44^, which warrant further investigation. Consistent with our observations, HO-1 have also been shown to promote iron overload in beta-thalassemia, sickle cell disease, and anthracycline cardiotoxicity^45–47^. Moreover, transgenic overexpression of HO-1 promoted iron accumulation in the brain^48^. Of note, although HO-1 is commonly regarded as a cytoprotective enzyme^49^, recent studies indicate that HO-1 can also exert detrimental effects^50^. Moreover, both pro- and anti-ferroptotic roles of HO-1 have been observed depending on cell types and pathological conditions^50^. To explain this discrepancy, accumulating evidence suggests that moderate activation of HO-1 elicits a cytoprotective effect whereas excessive and/or prolonged activation of HO-1 increases labile Fe^2+^, leading to ferroptotic cell death^50^. Importantly, a recent study by Miyamoto et al. showed that HO-1 silencing prevented sI/R-induced ferroptosis in cardiomyocytes^51^. Moreover, inactivation of HO-1 with ZnPP also attenuated doxorubicin-induced cardiac ferroptosis and cardiotoxicity^47^. Therefore, these findings support a detrimental role of HO-1 activation in the settings of sI/R and doxorubicin insults, where HO-1 is markedly upregulated, by promoting ferroptosis of cardiomyocytes.

Intriguingly, the upregulation of HO-1 in cardiomyocytes in response to oxidative stress was associated with the downregulation of Bach1, a transcriptional repressor of HO-1^34^. HO-1 is positively regulated by the transcription factor nuclear factor erythroid 2-related factor 2 (NRF2), but negatively regulated by Bach1^52^. Notably, inactivation of Bach1 is a prerequisite for HO-1 induction, which is dominant over NRF2-mediated HO-1 transcription^53^. Moreover, Bach1 inactivation can induce HO-1 expression without NRF2 nuclear accumulation^53^. Consistent with this notion, our results indicate that the primary event leading to HO-1 induction in response to oxidative stress is Bach1 downregulation. Importantly, deletion of Bach1 was sufficient to induce HO-1 expression in cardiomyocytes. Moreover, forced overexpression of Bach1 inhibited oxidative stress-induced ferroptosis. Together, these data suggest that Bach1-HO-1 signaling critically regulates oxidative stress-induced ferroptosis.

This study also reveals that mitochondria play a key role in oxidative stress-induced ferroptosis in cardiomyocytes. The role of mitochondria in ferroptosis has been controversial^35–38^. For instance, it has been shown that depletion of mitochondria had no effect on RLS3-induce ferroptosis in HT-1080 cells^35^. In contrast, subsequent studies found that mitochondrial DNA depletion or mitochondrial ROS quenching inhibited RSL3-induced ferroptosis^37, 38^. Moreover, mitochondria depletion prevented ferroptosis induced by cysteine-deprivation or Erastin^36^. Here we showed that mitochondrial free iron levels as well as lipid peroxidation were markedly elevated following oxidative stress. Moreover, targeted inhibition of mitochondrial iron overload by overexpressing FTMT markedly inhibited oxidative stress-induced mitochondrial lipid peroxidation and ferroptosis. Targeted inhibition of mitochondrial ROS, using mitoQ, SKQ1, or mCAT, also largely inhibited ferroptosis. These results suggest that mitochondrial iron overload and lipid ROS accumulation play an important role in oxidative stress-induced ferroptosis in cardiomyocytes. Of note, mitochondrial permeability transition pore (mPTP) has been implicated in oxidative stress-induced necrosis as well as ischemia/reperfusion injury^7^. However, Dixon et al. demonstrated that ferroptosis is independent of mPTP, since ferroptosis is not affected by inactivation of CypD, a key component of mPTP^9^. Consistent with this, we found that ablation of *Ppif* (gene encoding CypD) had no effect on tBHP-induced cell death in MEFs. Moreover, inactivation of CypD with CsA also failed to inhibit tBHP-induced cell death in cardiomyocytes. Similar effects were obtained in H9c2 cardiomyoblasts^54^. In contrast, another study showed that *Ppif* deletion prevented tBHP-induced cell toxicity in a human pancreatic cell line^55^. These results suggest that tBHP may induce cell death though mPTP-dependent or -independent mechanisms depending on cell types and cellular contexts.

Importantly, we further identified HO-1 mitochondrial translocation as a key mechanism for oxidative stress-induced mitochondrial iron overload and ROS accumulation. Our data suggest that oxidative stress promotes HO-1 translocation from the cytosol into mitochondria where it catalyzes heme degradation and iron release. In support of this notion, deletion of HO-1 abrogated oxidative stress-induced mitochondrial iron overload. Notably, HO-1 mitochondrial translocation has also been detected in several cell types in response to hypoxia, leading to mitochondrial ROS accumulation and dysfunction^56^. The precise mitochondria targeting sequence of HO-1 has not been identified^56^. It is possible that a cryptic mitochondria-targeting signal may exit which might be activated by oxidative stress^56^. Therefore, the mechanism of HO-1 mitochondrial translocation warrants further investigation. Importantly, overexpressing FTMT, a mitochondrial iron chelating protein, largely inhibited oxidative stress-induced ferroptosis, further linking mitochondria iron overload to ferroptosis. Consistent with our findings, a recent study on the dihydroorotate dehydrogenase (DHODH)-mediated mitochondria ferroptosis defense system also points to the role of mitochondrial iron in ferroptosis^57^. Moreover, mice with heart-specific overexpression of ABCB8, which exports iron out of the mitochondria, were more resistant to DOX cardiotoxicity, although the role of ABCB8 in ferroptosis has not been directly investigated^58^. Of note, other potential mechanisms may also contribute to mitochondria iron overload in ferroptosis. For example, increased mitochondrial iron uptake through iron transporters, such as mitoferrin-2, can mediate mitochondrial iron overload^59, 60^. Moreover, in photodynamic therapy-induced ferroptosis, cytosolic iron is translocated into mitochondria via the mitochondrial Ca^2+^ and Fe^2+^ uniporter (MCU), leading to mitochondrial iron overload. Nonetheless, these results highlight a key role of mitochondrial iron in mediating ferroptosis, suggesting that targeting mitochondrial iron overload may represent a new strategy for preventing ferroptosis. Whether mitochondrial iron overload mediates oxidative stress-induced ferroptosis in the heart in vivo warrants further investigation. Indeed, it has been shown that mitochondrial iron levels are elevated in the heart subjected to ischemia/reperfusion injury and pressure overload^61, 62^. Moreover, HO-1 is also upregulated in the heart under these pathological conditions. It will be important to further investigate the role of HO-1 mediated mitochondrial iron overload in cardiac ferroptosis in vivo and the physiological implications of this mechanism in the pathogenesis of oxidative stress-induced heart disease.

In summary, the present study identified ferroptosis as the major form of cardiomyocyte death triggered by organic oxidants-induced oxidative stress, in contrast to previous studies implicating other forms of cell death in this process, such as apoptosis and necroptosis. We also provide mechanistic evidence that oxidative stress induces cardiomyocyte ferroptosis by promoting lipid peroxidation via GSH depletion and GPX4 inactivation as well as iron overload through Bach1-HO-1 signaling. Moreover, we identified HO-1 mitochondrial translocation as a novel mechanism mediating mitochondrial iron overload and ROS accumulation. Targeted inhibition of mitochondrial iron overload or ROS accumulation effectively inhibited oxidative stress-induced ferroptosis. Therefore, targeting Bach1-HO-1 signaling and mitochondrial iron overload may serve as potential cytoprotective strategies in pathological conditions associated with oxidative stress.

### Supplementary Information

Supplementary Information.

## Data Availability

All data generated or analyzed during this study are either included in this manuscript or available from the corresponding author upon reasonable request.
