# Supplementary material for: Oxidative stress induces mitochondrial iron overload and ferroptotic cell death
Source: Sci Rep. 2023 Sep 19;13:15515. doi: 10.1038/s41598-023-42760-4 (PMC10509277; doi:10.1038/s41598-023-42760-4)

Supplemental Figure 1

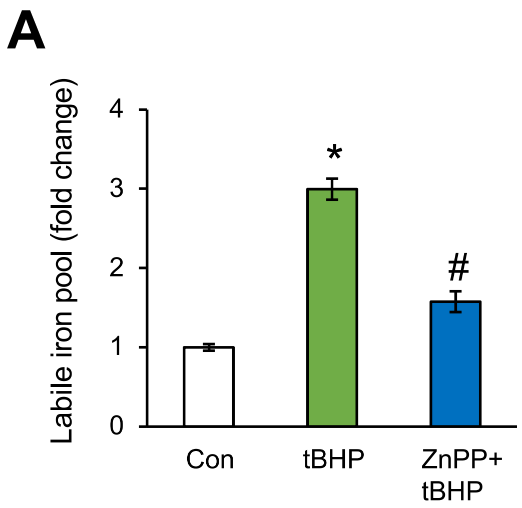

Supplemental Figure 1. Inactivation of HO-1 reduced tBHP-induced labile iron levels and cell death in cardiomyocytes. A. Assessment of labile iron pool in neonatal rat cardiomyocytes treated with tBHP in the presence and absence of ZnPP for 8 h. **B**. Quantification of cell death from cells treated as in A for 12 h. *$P < 0.05$ vs Control; #$P < 0.05$ vs tBHP. n = 3.

Supplemental Figure 2

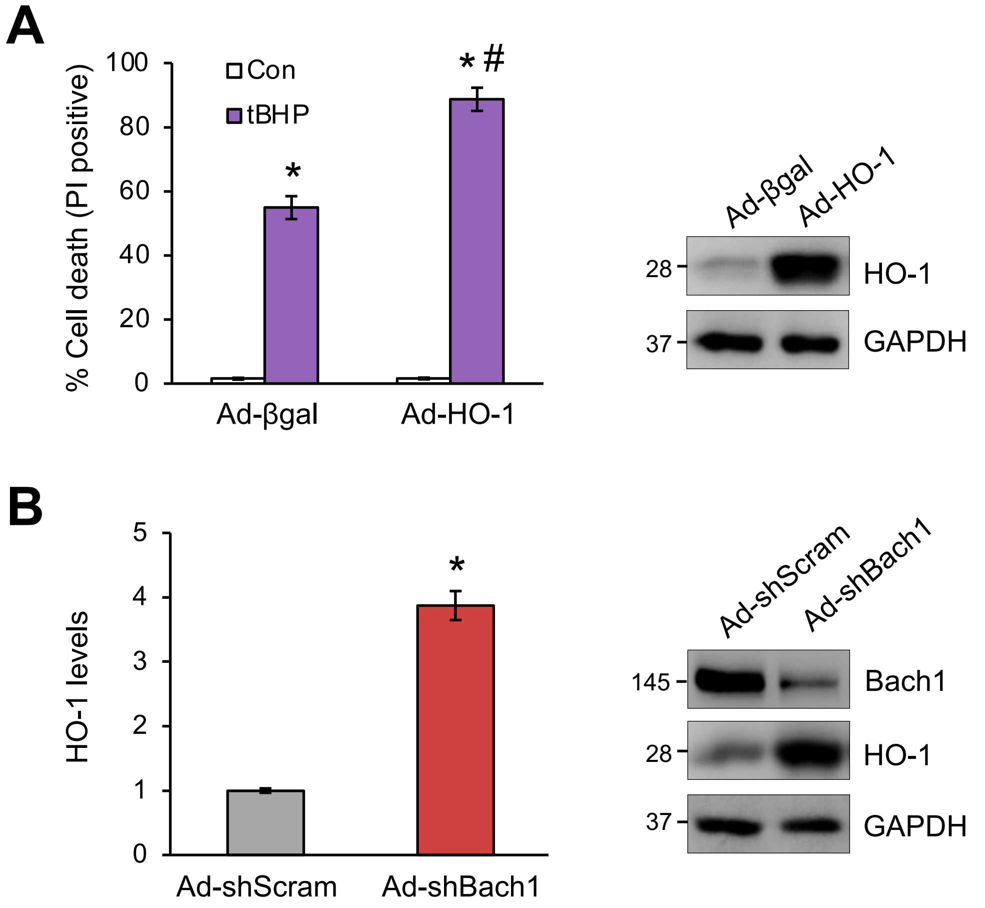

Supplemental Figure 2. Overexpression of HO-1 promoted tBHP-induced cell death in cardiomyocytes. A. Cell death was assessed from cardiomyocytes infected with Ad-βgal or Ad-HO-1 for 24 h followed by treatment with tBHP or vehicle control for 12 h. *P*< 0.05 vs Control; #*P*< 0.05 vs Ad-βgal + tBHP. n = 3. **B**. HO-1 expression in cardiomyocytes infected with adenoviral vectors for Bach1 shRNA (Ad-shBach1) or scrambled control (Ad-shScram) for 24 h. *P*< 0.05 vs Ad-shScram. n = 3.

Supplemental Figure 3

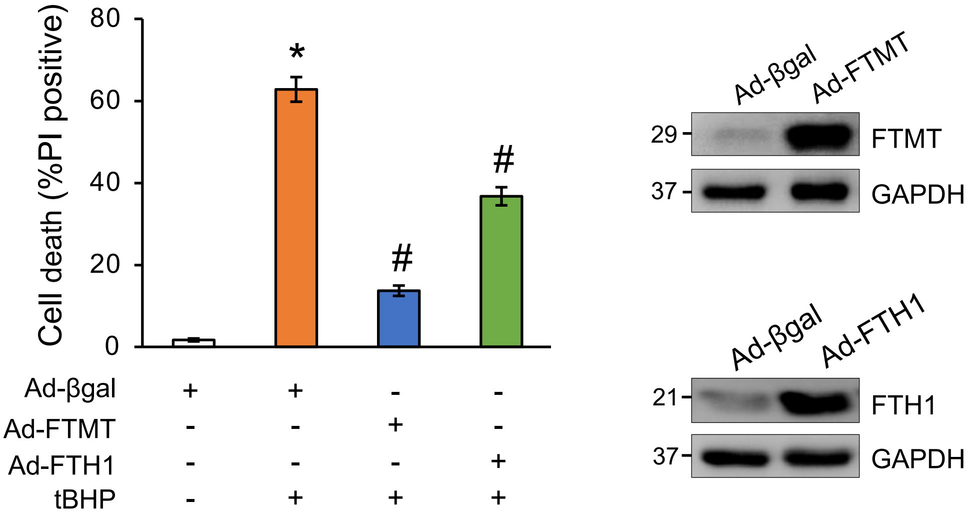

Supplemental Figure 3. Overexpression of mitochondrial ferritin (FTMT) inhibited tBHP-induced cell death in cardiomyocytes. Cell death was assessed from cardiomyocytes infected with Ad-βgal, Ad-FTMT, or Ad-FTH1 for 24 h followed by treatment with tBHP or vehicle control for 12 h. Ad-FTH1, adenovirus expressing ferritin heavy chain-1. FTMT and FTH1 expression levels were assessed by Western blotting. *P*< 0.05 vs Ad-βgal; #*P*< 0.05 vs Ad-βgal + tBHP. n = 3.

Supplemental Figure 4

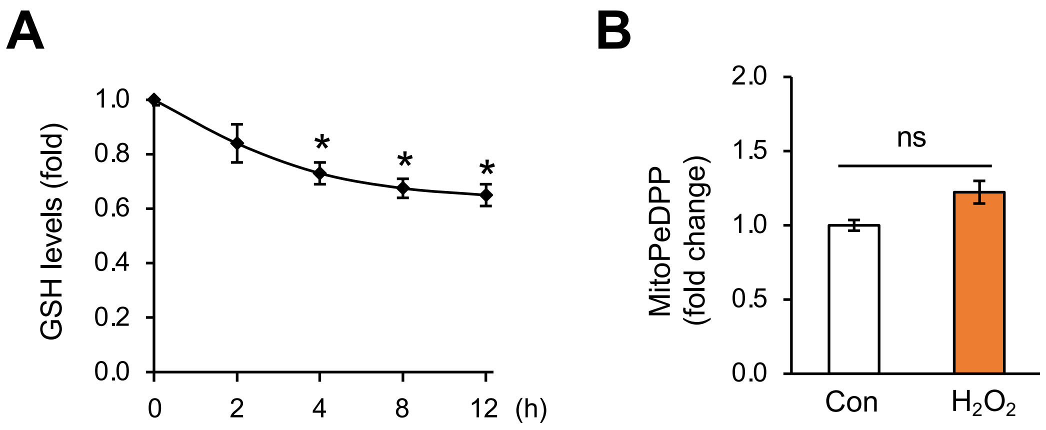

Supplemental Figure 4. Effect of H_2O_2 on GSH levels and mitochondrial lipid peroxidation in cardiomyocytes. A. GSH levels in cardiomyocytes treated with 200 μM H_2O_2 for the indicated time periods. **P*< 0.05 vs 0 h. n = 3. **B**. Mitochondrial lipid peroxidation assessed with MitoPeDPP in cardiomyocytes treated with 200 μM H_2O_2 for 8 h. ns, non-significant. n = 3.

Full length blots

Figure 1C

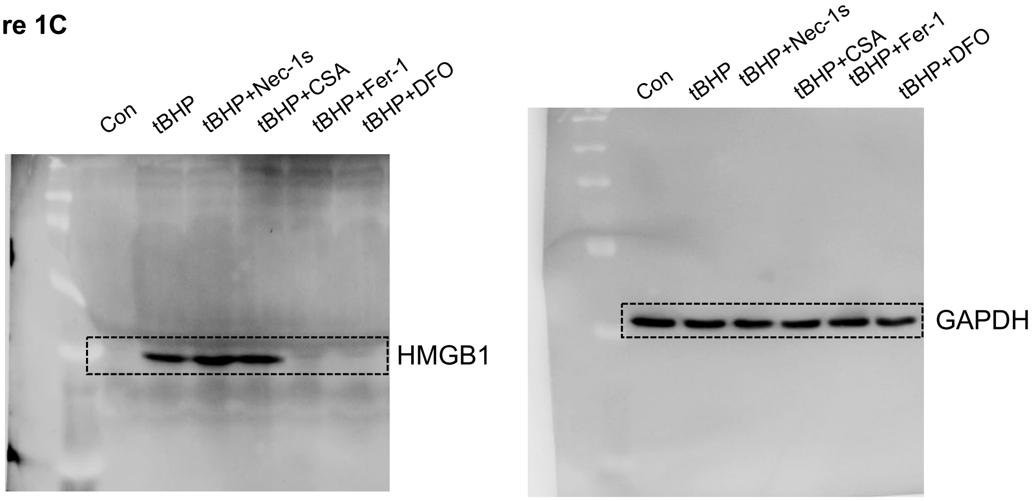

Figure 1F

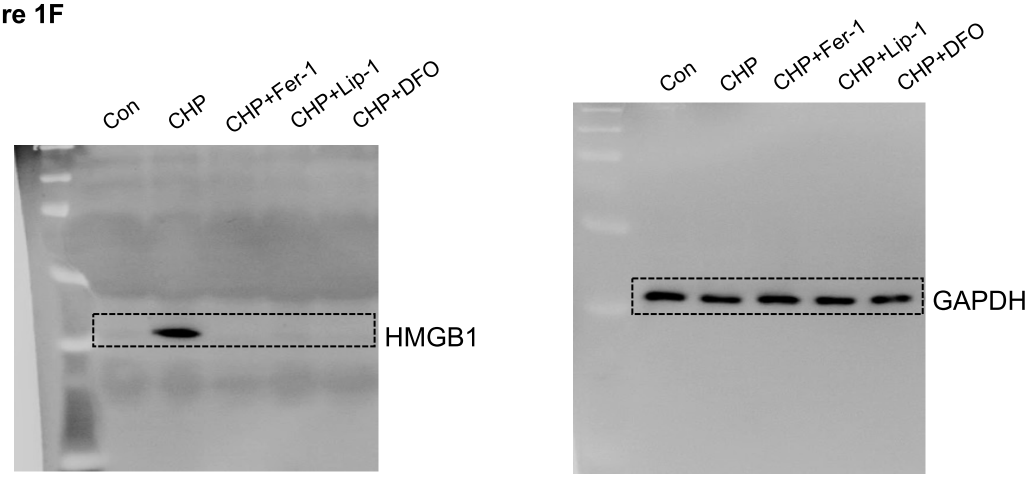

Figure 1H

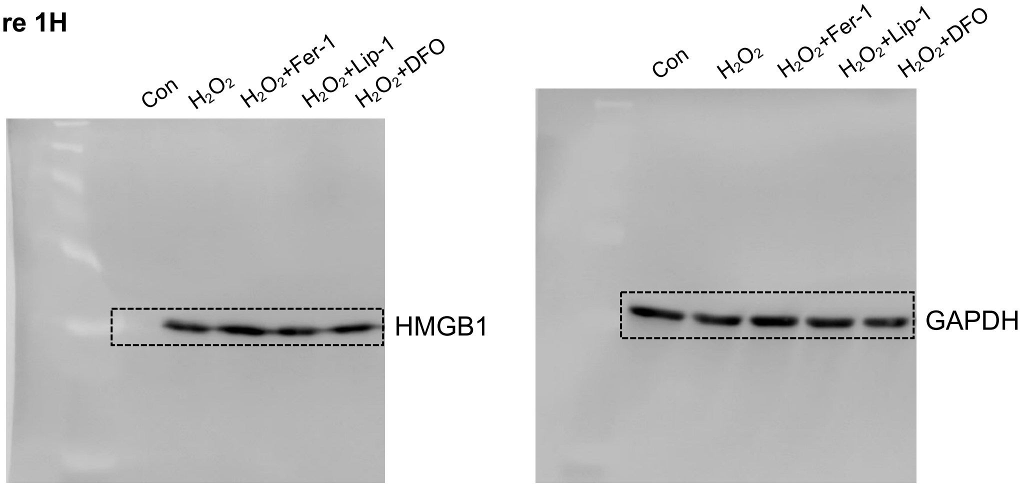

Figure 2D

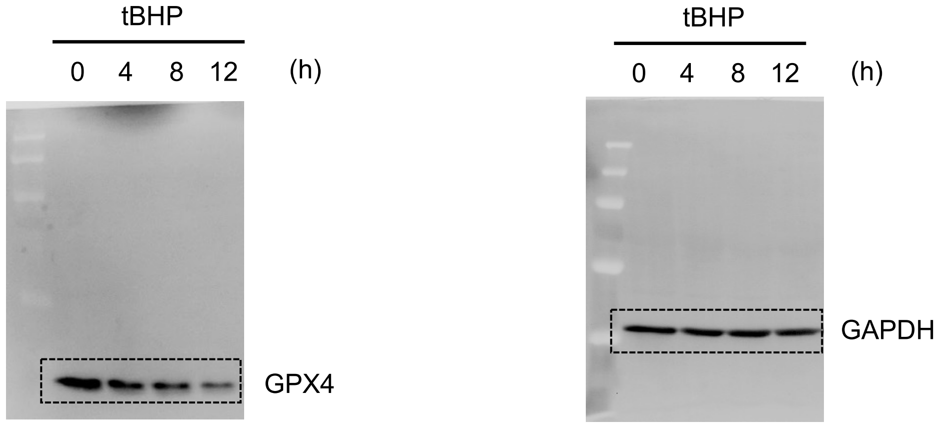

Figure 2F

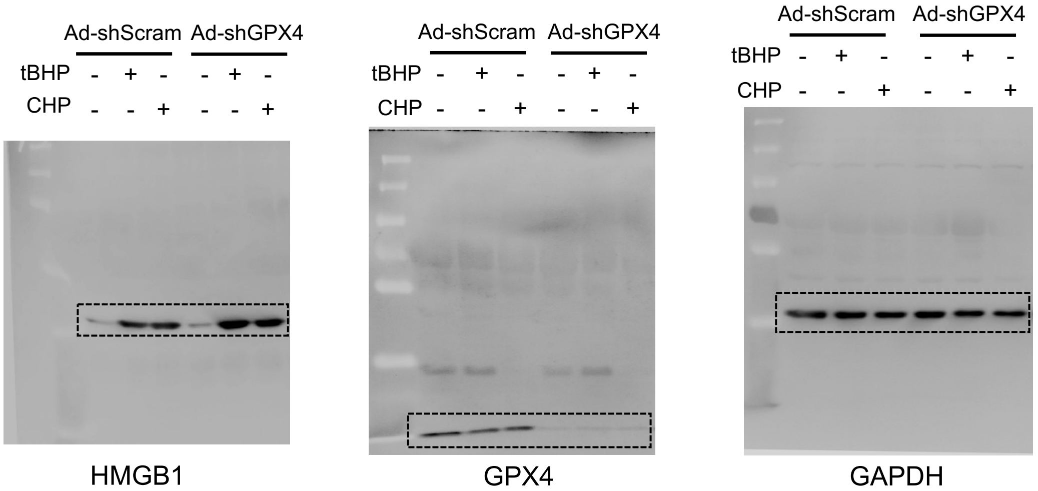

Figure 2H

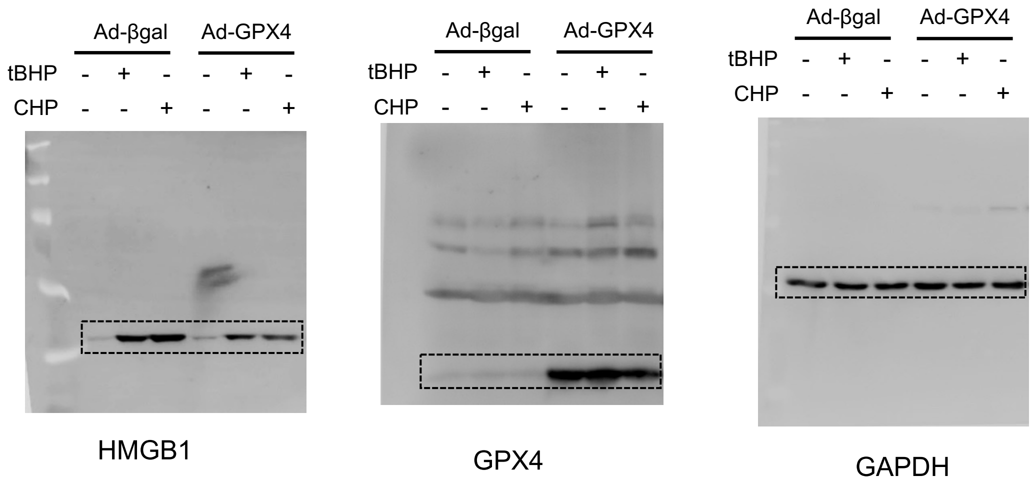

Figure 3B

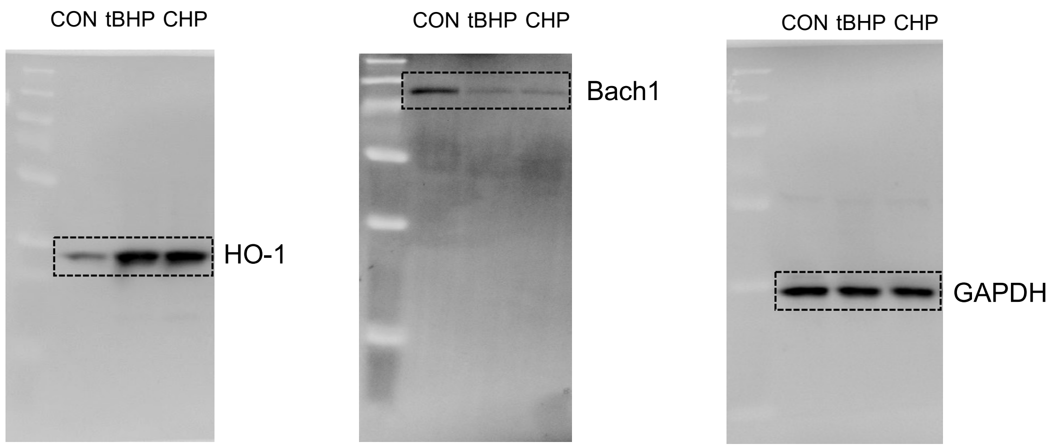

Figure 3C

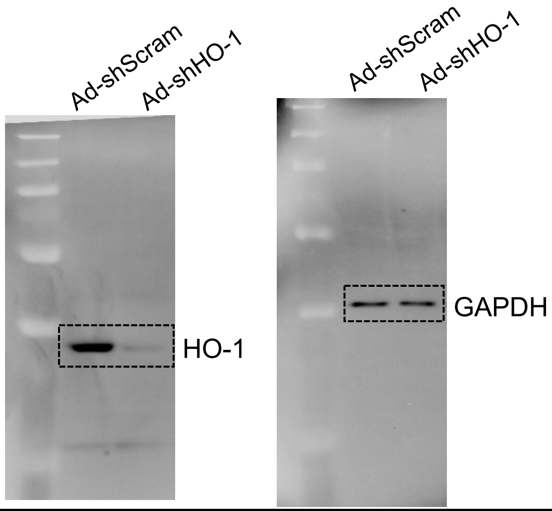

Figure 3F

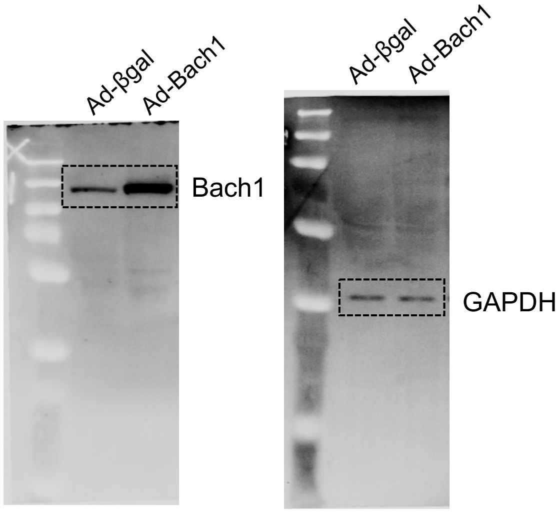

Figure 3E

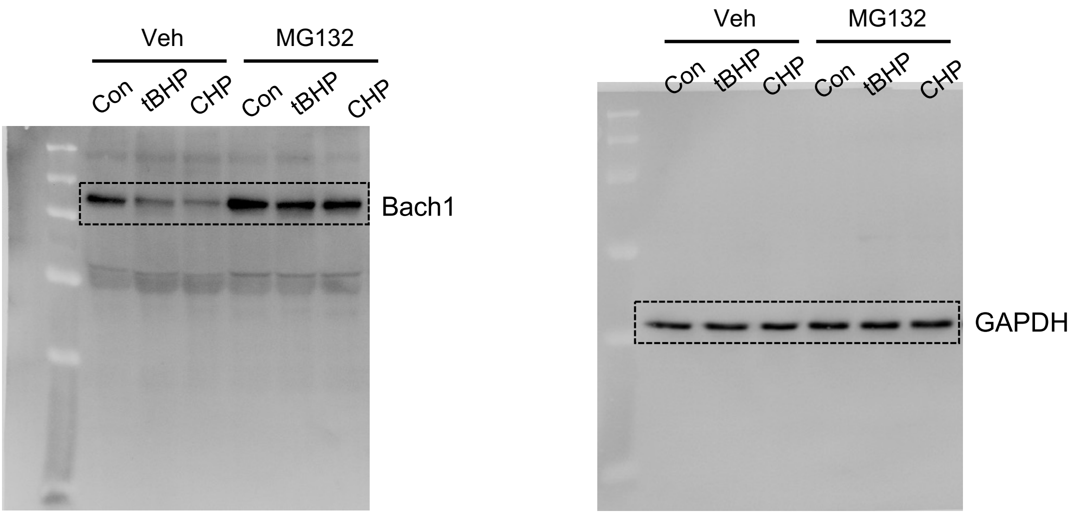

Figure 4B

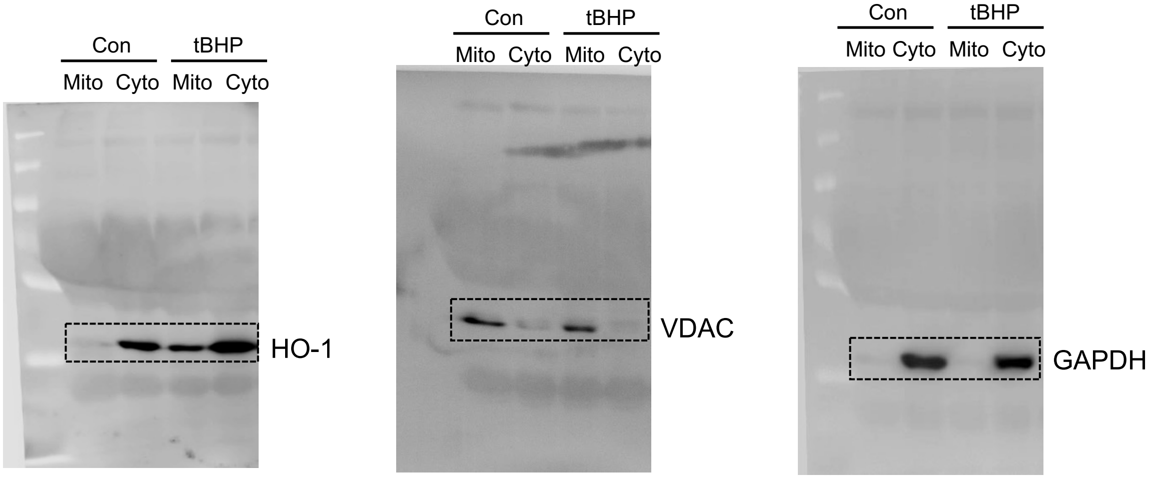

Figure 4D

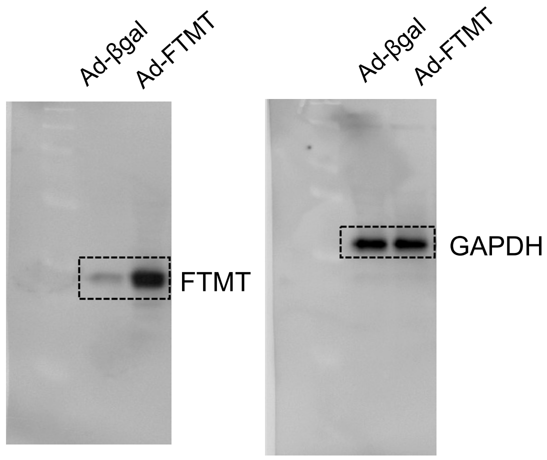

Figure 4E

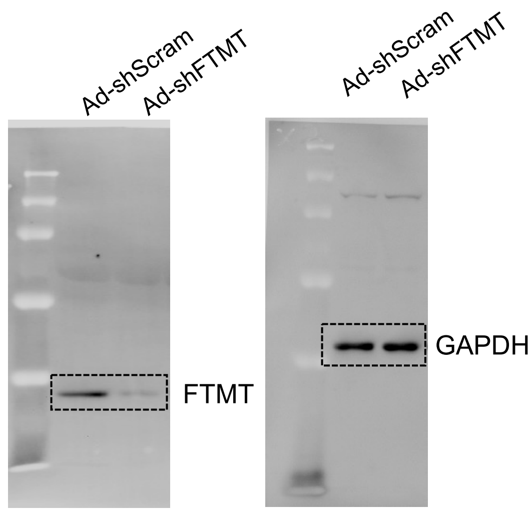

Figure 5E

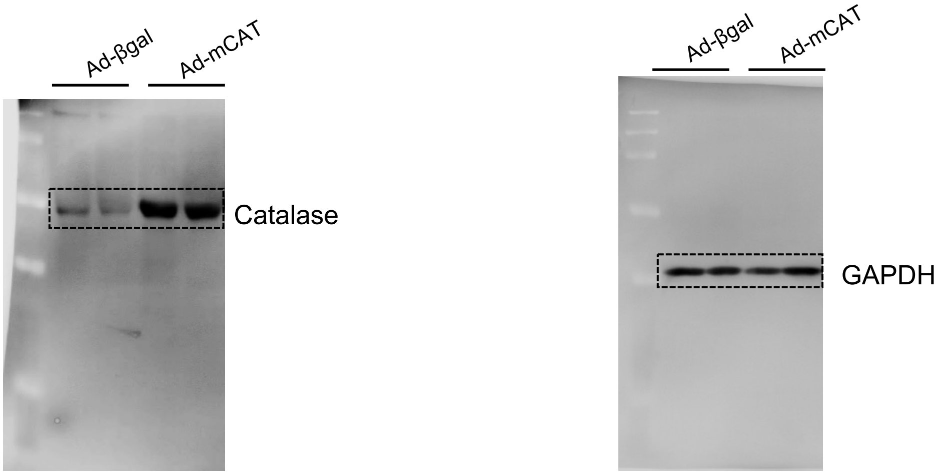

Figure 6E

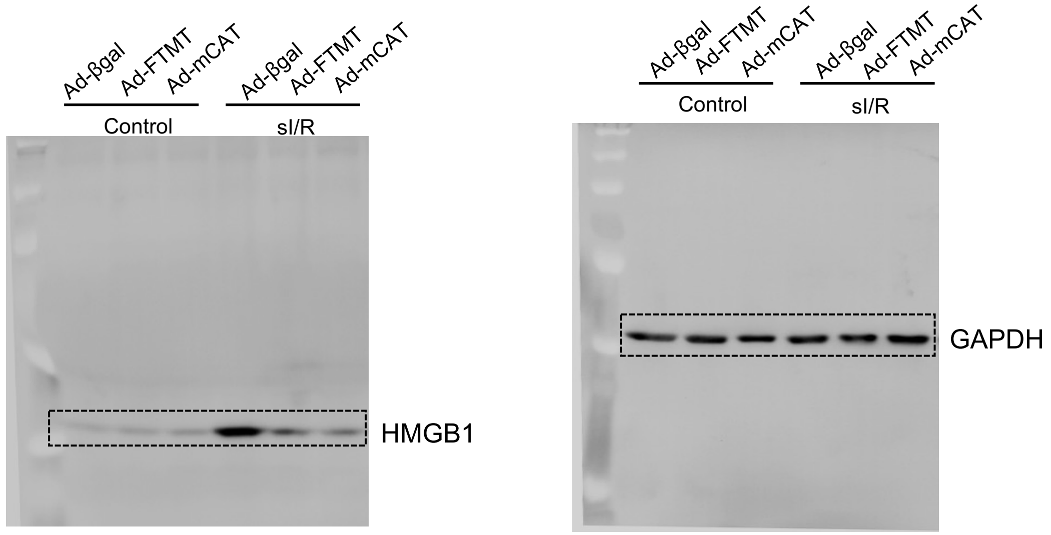

Figure 6H

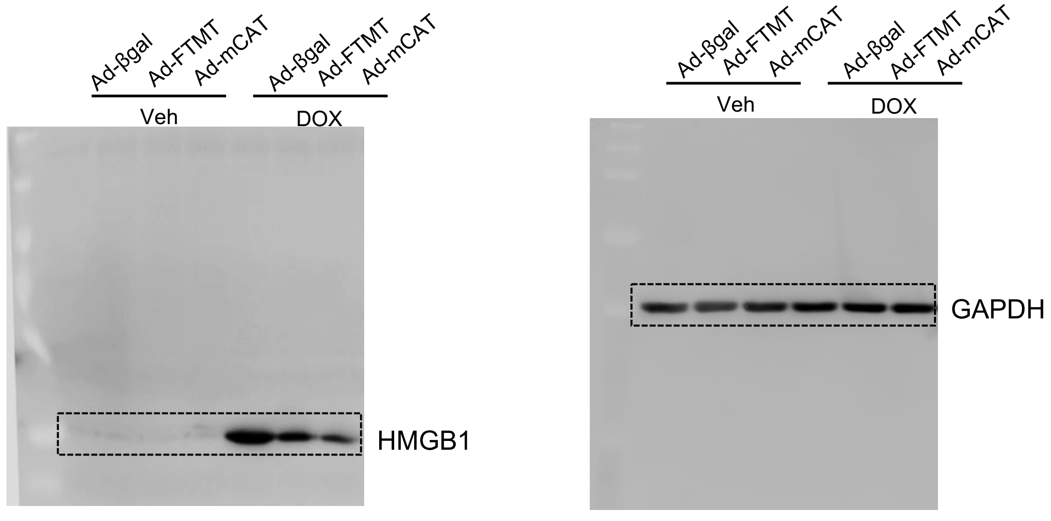

Suppl. Figure 2A

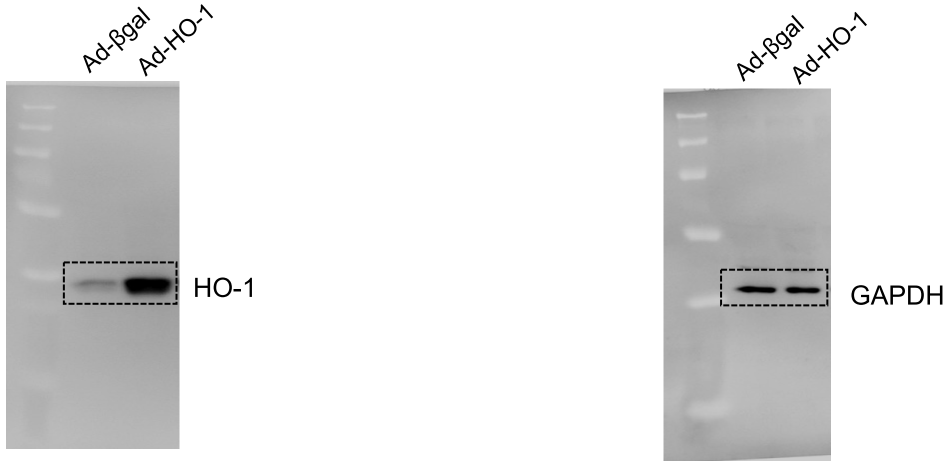

Suppl. Figure 2B

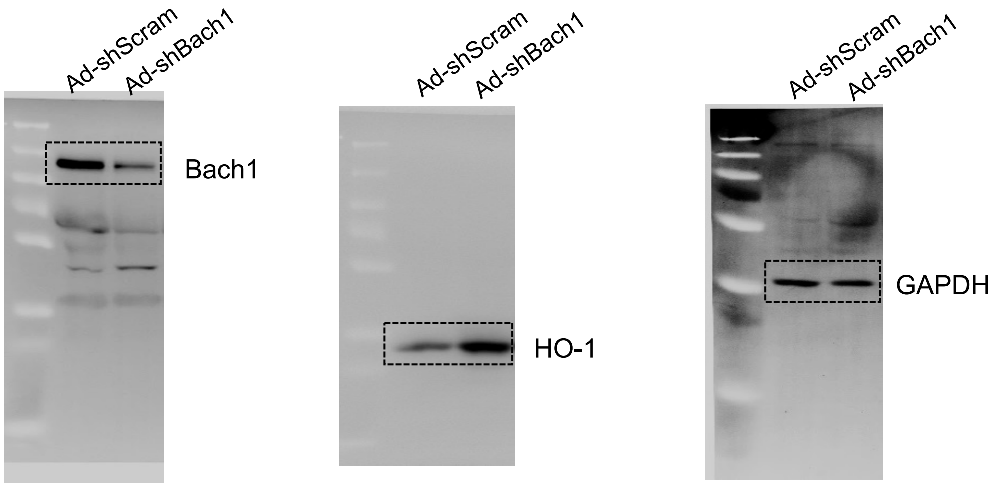

Suppl. Figure 3

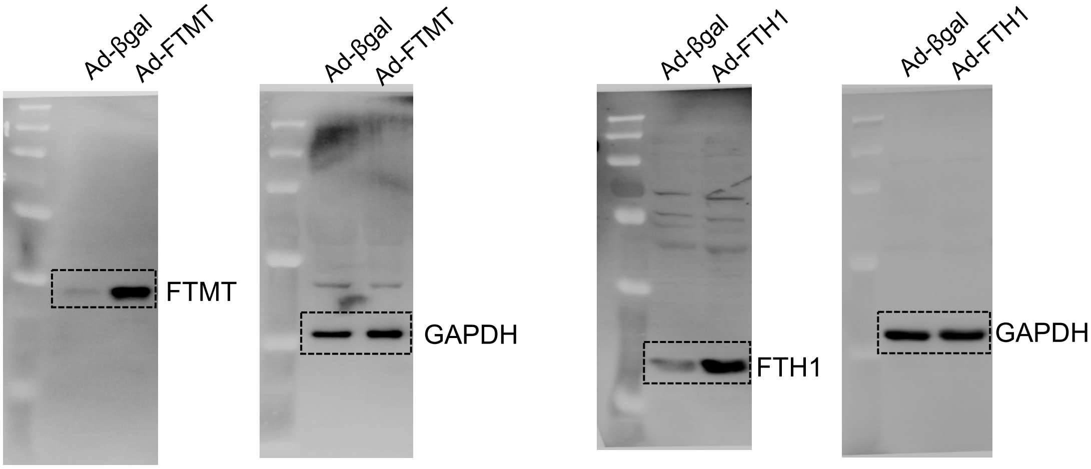

Supplement: Supplementary file 1 — Supplementary Information. [file 41598_2023_42760_MOESM1_ESM.pdf]
